# Sequential Tumor Microenvironment Reprogramming by Nanoplatform Potentiates Sonodynamic‐Chemodynamic Therapy and Immune Checkpoint Blockade in Breast Cancer

**DOI:** 10.1002/advs.202512135

**Published:** 2025-10-24

**Authors:** Yang Yu, Zheming Song, Anni Zhu, Jingchao Li, Rujia Fan, Bing Xiao

**Affiliations:** ^1^ Department of Breast Surgery Henan Provincial People's Hospital Zhengzhou University People's Hospital Henan University People's Hospital Zhengzhou Henan 450003 China; ^2^ State Key Laboratory of Advanced Fiber Materials College of Biological Science and Medical Engineering Donghua University Shanghai 201620 China; ^3^ Department of Obstetrics and Gynecology Henan Provincial People's Hospital Zhengzhou University People's Hospital Henan University People's Hospital Zhengzhou Henan 450003 China; ^4^ Department of Radiology Sir Run Run Shaw Hospital Zhejiang University School of Medicine Hangzhou 310016 China

**Keywords:** breast cancer, immunotherapy, sonodynamic therapy, stimulus‐responsive nanoparticles, tumor microenvironment

## Abstract

The complex tumor microenvironment (TME) remains a major barrier to effective breast cancer therapy. A modular nanoplatform capable of sequentially reprogramming the TME through cascade actions and responsive therapeutic functions is developed to enhance breast cancer immunotherapy. A hybrid nanoparticle (MCC) containing manganese dioxide (MnO_2_), calcium peroxide (CaO_2_), and chlorin e6 (Ce6) is synthesized and subsequently camouflaged with a tumor cell membrane. Surface conjugation of a PD‐L1 antibody (αP) is then achieved via a glutathione (GSH)‐responsive fragment, resulting in the formation of an integrated nanoplatform MCC@TM‐αP. Through dual‐targeting mechanisms involving the tumor cell membrane and the PD‐L1 antibody, MCC@TM‐αP achieves efficient enrichment at tumor sites. MCC@TM‐αP alleviates hypoxia by generating O_2_ from CaO_2_ in the acidic TME and scavenges GSH via the MnO_2_‐mediated Fenton‐like reaction, thereby markedly amplifying the sonodynamic efficacy of Ce6. The combined effects of sonodynamic therapy and chemodynamic therapy ablate tumors and reprogram the immunosuppressive TME. Upon cleavage of the GSH‐responsive fragment by intratumoral GSH, MCC@TM‐αP releases the PD‐L1 antibody, eliciting a robust immune response that eradicates metastatic tumors. In murine breast cancer models, this therapeutic strategy enhances tumor infiltration by effector T cells and suppresses metastatic progression. By sequentially decoupling the immunosuppressive mechanisms, this study provides a programmable approach to potentiate immunotherapy and overcome TME‐driven resistance.

## Graphical abstract

A modular nanoplatform capable of *sequentially reprogramming* tumor microenvironment via cascade actions and responsive therapeutic functions is reported for breast cancer therapy. Via a dual‐targeting mechanism and responsive PD‐L1 antibody release, this nanoplatform enables amplified sonodynamic‐chemodynamic therapy and elicits robust immune response for eradicating metastatic tumors. This therapeutic tool demonstrates good antitumor and anti‐metastasis effects in murine breast cancer models.

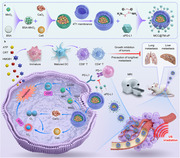

## 1 Introduction

Tumors represent a significant global health challenge and are characterized by complex pathogenesis involving intricate interactions among multiple cells and signaling pathways.^[^ ^1^, ^2^, ^3^ ^]^ During tumor initiation and progression, the tumor microenvironment (TME)—composed of tumor cells, immune cells, fibroblasts, vascular endothelial cells, the extracellular matrix, and signaling molecules—provides crucial support for tumor growth and metastasis.^[^ ^4^, ^5^, ^6^ ^]^ The immunosuppressive TME is a central factor mediating tumor immune escape and plays a pivotal role in limiting the efficacy of current immunotherapies. Characterized by hypoxia, elevated glutathione (GSH) levels, and immunosuppressive components, the TME creates formidable barriers to effective treatment, including limited drug penetration and adaptive therapy resistance.^[^ ^7^, ^8^, ^9^ ^]^ It not only inhibits the activation and infiltration of effector T cells but also disrupts antigen‐presenting cell function, leading to limited response rates to strategies such as immune checkpoint blockade (ICB). Hypoxia promotes tumor aggressiveness and suppresses immune cell function, whereas excessive GSH scavenges reactive oxygen species (ROS), diminishing the efficacy of ROS‐based therapies.^[^ ^10^, ^11^, ^12^ ^]^ Conventional strategies usually fail to address these multifaceted challenges; therefore, innovative approaches capable of extensively remodeling the TME are needed for enhancing treatment efficacy.

Immunotherapy has emerged as a novel treatment strategy, demonstrating remarkable potential to effectively eliminate locally advanced tumors while suppressing distant metastasis and recurrence.^[^ ^13^, ^14^, ^15^ ^]^ ICB therapy represents a major approach in cancer immunotherapy.^[^ ^16^, ^17^, ^18^ ^]^ However, a significant proportion of tumors exhibit poor responses to ICB owing to insufficient tumor‐infiltrating immune cells or an immunosuppressive TME. Clinically, immunotherapy faces two major challenges: 1) the overall response rate remains low, with only ≈10–30% of patients showing an effective response to ICB;^[^ ^19^, ^20^, ^21^ ^]^ and 2) immunotherapy frequently induces various immune‐related adverse events, particularly in the context of high‐dose administration and combination immunotherapies.^[^ ^22^, ^23^, ^24^ ^]^ These limitations underscore the urgent need to develop safe and effective strategies to achieve optimal antitumor immunity.

The biological applications of nanomaterials have opened new therapeutic avenues for cancer treatment.^[^ ^25^, ^26^, ^27^ ^]^ Functional nanosystems with optimized drug delivery profiles exhibit considerable potential in cancer therapy by enhancing tumor penetration and accumulation and by reprogramming the TME.^[^ ^28^, ^29^, ^30^ ^]^ In particular, stimuli‐responsive nanoplatforms capable of precisely delivering therapeutic agents and remodeling the TME to improve therapeutic outcomes have been demonstrated.^[^ ^31^, ^32^, ^33^, ^34^, ^35^ ^]^ Among these, nanoplatforms that integrate sequential and cascade actions hold great promise for TME reprogramming.^[^ ^36^, ^37^, ^38^ ^]^ For instance, manganese dioxide (MnO_2_)‐based nanoparticles can alleviate hypoxia by catalyzing the conversion of endogenous H_2_O_2_ into O_2_, while simultaneously depleting GSH to enhance ROS‐mediated therapies.^[^ ^39^, ^40^, ^41^, ^42^ ^]^ Moreover, nanoplatforms serve as versatile systems for integrating different therapeutic modalities to achieve combinational or synergistic effects, thereby triggering tumor immunogenic cell death (ICD) and remodeling immunosuppressive components.^[^ ^43^, ^44^, ^45^, ^46^ ^]^ However, most existing nanoplatforms focus on single aspects of the TME and fail to achieve comprehensive reprogramming.^[^ ^47^, ^48^, ^49^, ^50^ ^]^ A sequential and multiple strategy that simultaneously addresses hypoxia, redox imbalance, and immunosuppression can improve therapeutic outcomes by creating a more permissive environment for immunotherapy.^[^ ^51^, ^52^, ^53^, ^54^ ^]^

**Figure 1:**
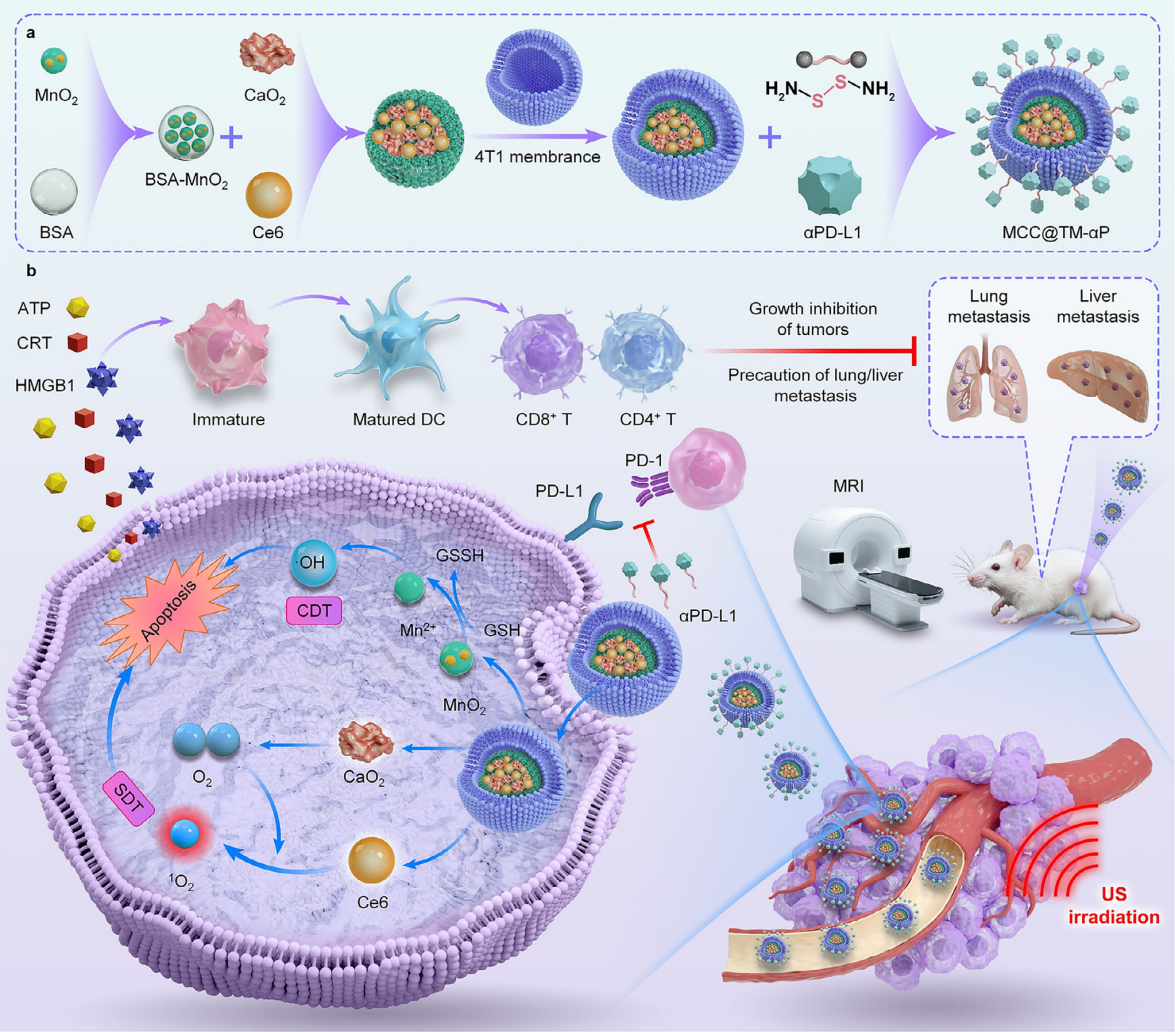
Design of a biomimetic and multifunctional nanoplatform (MCC@TM‐αP) for sequential TME reprogramming and enhanced breast cancer immunotherapy. a) Schematic illustration of the construction procedure of MCC@TM‐αP. b) Schematic representation of sequential TME reprogramming and combined sonodynamic therapy (SDT), chemodynamic therapy (CDT), and immunotherapy of breast cancer using MCC@TM‐αP.

We herein developed a biomimetic and multifunctional nanoplatform (MCC@TM‐αP) for sequential TME reprogramming and enhanced breast cancer immunotherapy. MCC@TM‐αP consists of MnO_2_, calcium peroxide (CaO_2_), and chlorin e6 (Ce6) encapsulated within a nanoparticle camouflaged with a tumor cell membrane (TM) for homologous targeting, followed by surface conjugation of a PD‐L1 antibody (αP) via a GSH‐cleavable linker (**Figure** **1**a).^[^ ^55^, ^56^ ^]^ The dual‐targeting mechanism mediated by the TM and PD‐L1 antibody ensures efficient tumor accumulation. Upon reaching the TME, MCC@TM‐αP alleviates hypoxia through O_2_ generation from CaO_2_ and depletes GSH via MnO_2_‐mediated redox reactions, thereby amplifying both sonodynamic therapy (SDT) (mediated by Ce6 under ultrasound irradiation) and chemodynamic therapy (CDT) effects. The combination of SDT and CDT induced potent tumor cell ablation while reprogramming the immunosuppressive TME (Figure 1b). GSH‐triggered release of the PD‐L1 antibody further enhanced T cell activity, establishing a robust immune response. In murine breast cancer models, this approach demonstrated significant primary tumor regression and inhibition of metastasis. Overall, this study presents a novel nanoplatform that integrates TME modulation, oxidative stress amplification, and immune checkpoint blockade into a single, sequential system, thereby providing a promising avenue for the treatment of aggressive and metastatic breast cancer.

## 2 Results and Discussion

### 2.1 Material Synthesis and Property Analysis

MnO_2_‐dotted BSA was synthesized following our previously reported procedures^[^ ^57^ ^]^ and was subsequently used to stabilize CaO_2_ and the sonosensitizer Ce6, forming a hybrid MnO_2_–CaO_2_–BSA–Ce6 (MCC) nanoparticle. After surface camouflaging with 4T1 tumor cell membranes, biomimetic nanoparticles (MCC@TM) were obtained. Subsequent conjugation of a PD‐L1 antibody (αP) onto the cell membrane using a GSH‐responsive fragment led to the construction of the final nanoplatform (MCC@TM‐αP).

**Figure 2:**
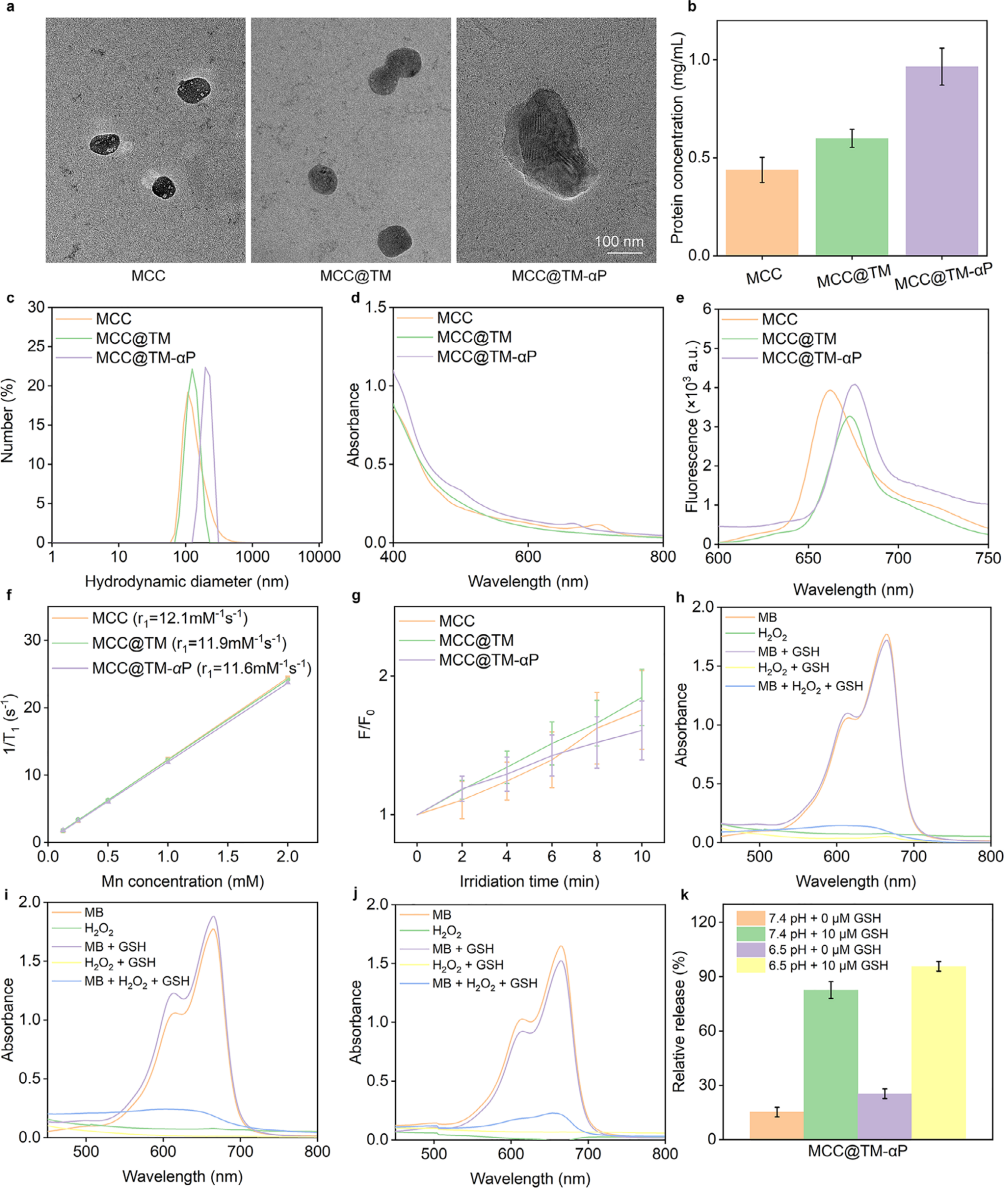
Material synthesis and property analysis. a) Size and morphology characterization of MCC, MCC@TM, and MCC@TM‐αP. b) Protein content analysis of MCC, MCC@TM, and MCC@TM‐αP (*n* = 3). c) Hydrodynamic diameter measurements of MCC, MCC@TM, and MCC@TM‐αP. d) UV–vis absorbance spectra of MCC, MCC@TM, and MCC@TM‐αP. e) Fluorescence signals of MCC, MCC@TM, and MCC@TM‐αP. f) r_1_ relaxivity measurements of MCC, MCC@TM, and MCC@TM‐αP. g) ^1^O_2_ generation assays for MCC, MCC@TM, and MCC@TM‐αP with exposure to US (*n* = 3). ·OH generation assays for h) MCC, i) MCC@TM, and j) MCC@TM‐αP. k) Relative αP release rates from MCC@TM‐αP under different conditions (*n* = 3).

MCC, MCC@TM, and MCC@TM‐αP exhibited a spherical morphology with diameters ranging from 120 to 200 nm, as confirmed using TEM (**Figure** **2**a). Protein content analysis showed the highest protein loading for MCC@TM‐αP, attributed to cell membrane camouflaging and αP conjugation, followed by MCC@TM, which involved only cell membrane coating (Figure 2b). Owing to their different surface modifications, the nanoparticles exhibited distinct hydrodynamic diameters: 125.6 nm for MCC, 133.1 nm for MCC@TM, and 197.6 nm for MCC@TM‐αP (Figure 2c). Their zeta potentials also differed, measuring −11.8, −17.4, and −3.4 mV for MCC, MCC@TM, and MCC@TM‐αP, respectively (Figure S1, Supporting Information). Particle size and zeta potential measurements were performed in ultrapure water at a sample concentration of 10 µg mL^−1^, with samples equilibrated for 2 min at 25 °C prior to analysis. Particle size data represent the intensity‐weighted average distributions from three independent measurements, whereas zeta potential values are presented as the mean ± standard deviation (SD) from three measurements. Notably, the hydrodynamic diameters of MCC, MCC@TM, and MCC@TM‐αP did not exhibit any significant increase over 14 d, confirming the absence of aggregation (Figure S2, Supporting Information). MCC, MCC@TM, and MCC@TM‐αP showed consistent absorbance profiles in the 400–800 nm wavelength range (Figure 2d). Owing to the presence of Ce6, a pronounced fluorescence signal was observed for MCC, MCC@TM, and MCC@TM‐αP at similar emission peaks, enabling their tracking in vitro and in vivo (Figure 2e).

The T_1_‐weighted magnetic resonance imaging (MRI) capabilities of MCC, MCC@TM, and MCC@TM‐αP were evaluated. All three exhibited concentration‐dependent grayscale imaging, with higher concentrations resulting in brighter signals. Their r_1_ relaxivities were consistent, ranging from 11.6 to 12.1 mM^−1 ^s^−1^ for these nanosystems (Figure 2f). The high r_1_ relaxivity of these nanoparticles can be attributed to their unique core–shell structure and the effective loading of MnO_2_, which enhance their interaction with water protons. These results demonstrate that the constructed nanosystem possesses excellent T_1_‐weighted MRI performance while enabling TME‐responsive diagnosis and therapy. Under US irradiation, efficient ^1^O_2_ production was confirmed as the fluorescence signal of ^1^O_2_ probe gradually increased, reaching ≈1.6–1.8 fold after 10 min, confirming the effective sonodynamic activity of MCC, MCC@TM, and MCC@TM‐αP, owing to the integration of the sonosensitizer Ce6 (Figure 2g). After incubation of MCC, MCC@TM, and MCC@TM‐αP with H_2_O_2_ and GSH, ·OH generation was confirmed by observing the absorbance changes of the ·OH indicator (Figure 2h–j). GSH‐triggered αP release was then confirmed, with the release rate reaching 95.6% at pH 6.5 in the presence of GSH, compared to less than 25.4% without GSH incubation (Figure 2k). These results demonstrate the responsive αP delivery by integrating the GSH‐responsive fragment into the nanosystems.

### 2.2 In Vitro Redox‐Induced Therapeutic and ICD Assays

**Figure 3:**
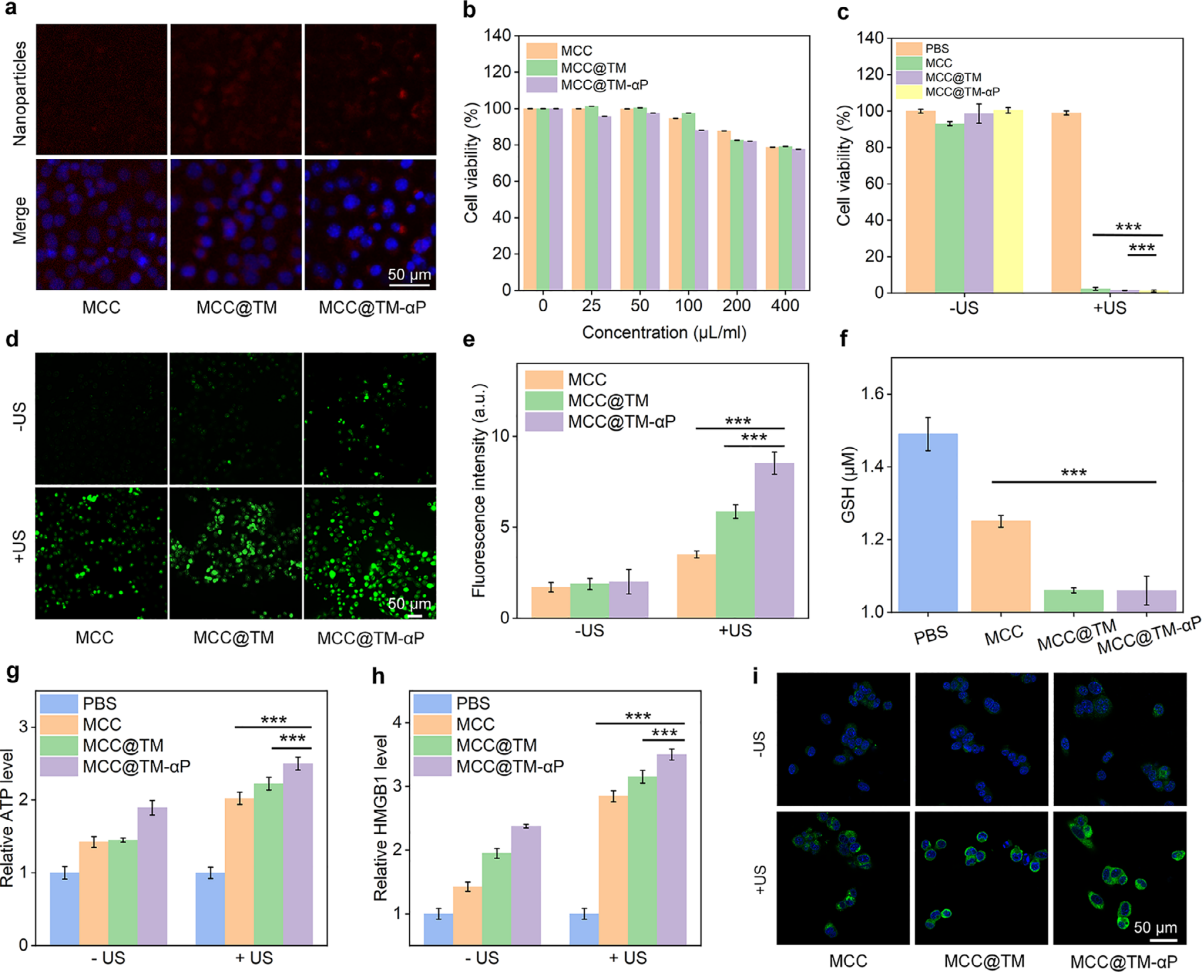
In vitro redox‐induced therapeutic and immunogenic cell death (ICD) assays. a) Fluorescence images of 4T1 cells for cellular internalization analysis of MCC, MCC@TM, and MCC@TM‐αP. b) Viability assay of 4T1 cells after incubation with MCC, MCC@TM, or MCC@TM‐αP (*n* = 5). c) Viability assay of 4T1 cells after incubation with MCC, MCC@TM, or MCC@TM‐αP incubation followed by US exposure (*n* = 5). d) Fluorescence images of 4T1 cells for reactive oxygen species (ROS) level assessment after incubation with MCC, MCC@TM, or MCC@TM‐αP. e) Quantification of ROS levels by measuring fluorescence signal intensity (*n* = 3). f) Glutathione (GSH) level assay in 4T1 cells after incubation with MCC, MCC@TM, or MCC@TM‐αP (*n* = 5). g) ATP assay in cells after incubation with MCC, MCC@TM, or MCC@TM‐αP and US exposure (*n* = 5). h) HMGB1 assay in cells after incubation with MCC, MCC@TM, or MCC@TM‐αP and US exposure (*n* = 3). i) Fluorescence images for CRT assay in cells after incubation with MCC, MCC@TM, or MCC@TM‐αP and US exposure. All data are presented as the mean ± standard deviation (SD) (^*^ *p*  < 0.05, ^**^ *p* <0.01, ^***^ *p*  < 0.001; two‐tailed Student's *t*‐tests).

4T1 breast cancer cells were used to assess in vivo cellular uptake and therapeutic effects. Owing to the dual‐targeting mechanism provided by tumor cell membrane camouflage and PD‐L1 antibody surface conjugation, MCC@TM‐αP exhibited the strongest fluorescence signal in 4T1 cells (**Figure** **3**a). Cells incubated with MCC@TM displayed a more pronounced signal than those treated with MCC alone, reflecting the enhanced homologous targeting conferred by the cell membrane camouflage. The fluorescence intensity in the MCC@TM‐αP group increased by 1.13‐ and 1.28‐fold compared with that in the MCC@TM and MCC groups, respectively (Figure S3, Supporting Information). These results confirmed the dual‐targeting capability of MCC@TM‐αP in facilitating nanoplatform internalization by tumor cells. After incubation with MCC, MCC@TM, and MCC@TM‐αP at concentrations of 25–200 µg mL^−1^, 4T1 cells exhibited cell viability values above 80% (Figure 3b), indicating good cytocompatibility. In vitro therapeutic assays further showed that 4T1 viability decreased to ≈2.3% following treatment with MCC, MCC@TM, and MCC@TM‐αP combined with US exposure (Figure 3c). This marked reduction in viability confirms the potent in vitro tumor cell‐killing effects.

Intracellular redox levels were subsequently investigated. Compared with the control group, ROS production was confirmed by fluorescence signals in cells treated with MCC, MCC@TM, or MCC@TM‐αP, which resulted from ·OH generation through the Fenton‐like reaction of MnO_2_ (Figure 3d). In the US‐irradiated groups following incubation with MCC, MCC@TM, or MCC@TM‐αP, markedly stronger ROS signals were observed, owing to the combined production of ·OH by MnO_2_ and ^1^O_2_ through the sonodynamic effect of Ce6. Notably, the ROS signal intensity in the MCC@TM‐αP + US group was ≈2.4‐ and 1.5‐fold higher than that in the MCC + US and MCC@TM + US groups (Figure 3e). The targeting capability and enhanced cellular internalization of MCC@TM‐αP contributed to the elevated ROS production. In comparison with the PBS group, GSH levels in cells treated with MCC, MCC@TM, or MCC@TM‐αP showed a significant decline (Figure 3f), as GSH was scavenged by MnO_2_.

After incubation with MCC, MCC@TM, or MCC@TM‐αP, followed by US exposure, biomarkers associated with ICD were assessed. In MCC‐, MCC@TM‐, and MCC@TM‐αP‐treated cells, ATP secretion increased by 1.4‐, 1.5‐, and 1.9‐fold, respectively, compared with that in control cells (Figure 3g). ATP levels were further elevated to 2.0‐, 2.2‐, and 2.5‐fold in the MCC + US, MCC@TM + US, and MCC@TM‐αP + US groups, respectively. Extracellular HMGB1 release levels also significantly increased upon treatment with the nanoplatforms alone (1.4‐fold for MCC, 1.9‐fold for MCC@TM, and 2.4‐fold for MCC@TM‐αP) and in combination with US exposure (2.8‐fold for MCC + US, 3.2‐fold for MCC@TM + US, and 3.5‐fold for MCC@TM‐αP + US) (Figure 3h). Concurrently, increased surface CRT exposure was observed in all treated groups, with the MCC@TM‐αP + US group showing particularly strong CRT signals (Figure 3i). Compared with the control group, CRT signal intensity was significantly elevated across treatment groups, reaching a fivefold increase in the MCC@TM‐αP + US group (Figure S4, Supporting Information). Collectively, these findings demonstrate the induction of ICD, which can promote antitumor immunity.

### 2.3 In Vivo Tumor Enrichment and MRI Performance

**Figure 4:**
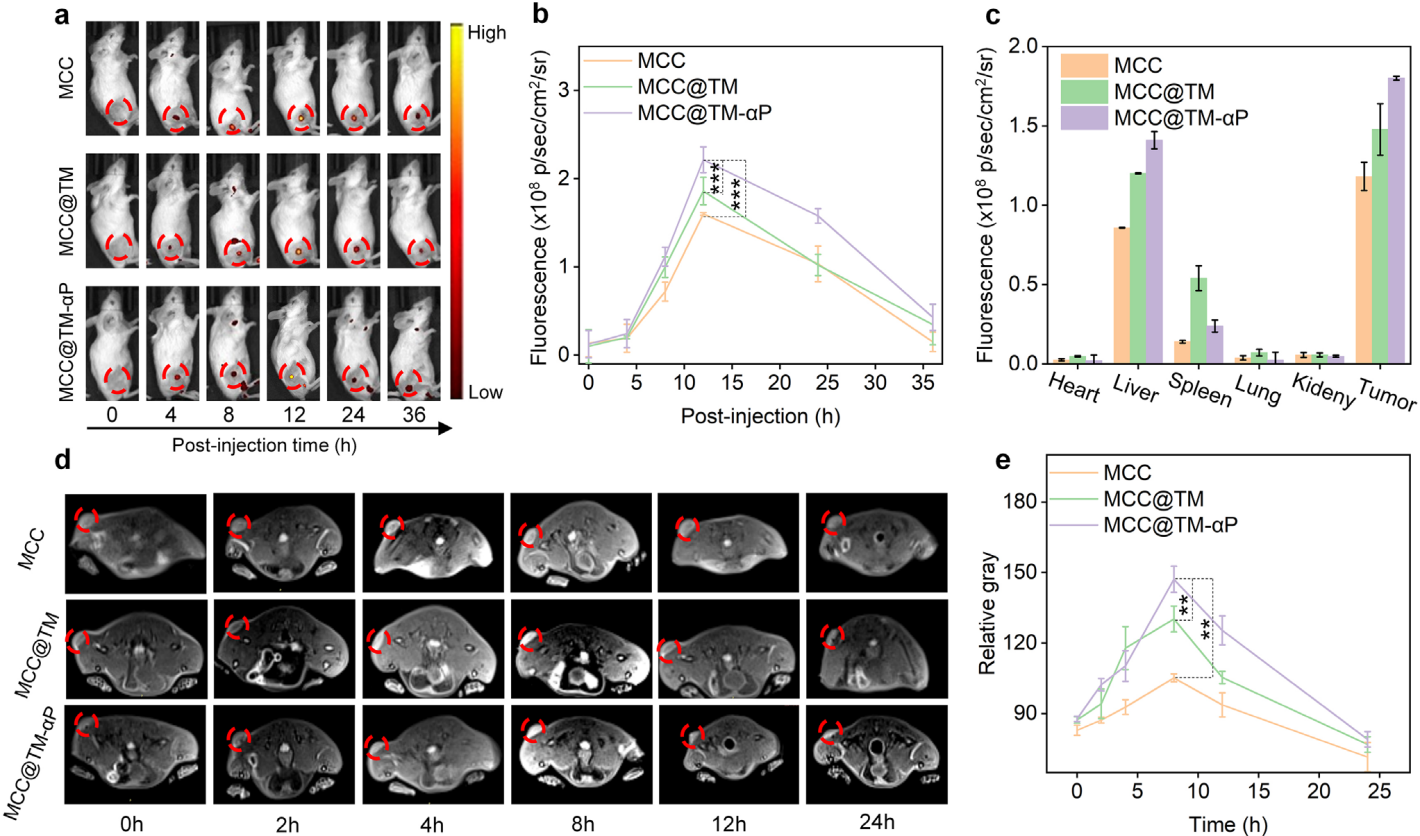
In vivo tumor enrichment and MRI performance. a) Tumor accumulation analysis of MCC, MCC@TM, and MCC@TM‐αP following tail vein injection. b) Quantitative analysis of tumor fluorescence signals after MCC, MCC@TM, and MCC@TM‐αP injection (*n* = 3). c) Biodistribution analysis of MCC, MCC@TM, and MCC@TM‐αP via tail vein injection. d) T_1_‐weighted MR images of tumors in mice after tail vein injection of MCC, MCC@TM, or MCC@TM‐αP. e) Quantitative analysis of tumor signal intensity in T_1_‐weighted MR images (*n* = 3). All data are presented as the mean ± SD (^*^ *p* <0.05, ^**^ *p*  < 0.01, ^***^ *p*  < 0.001; two‐tailed Student's *t*‐tests).

The tumor‐targeting capabilities of the nanoplatforms were systematically evaluated in murine breast cancer models via tail vein injection. Fluorescence imaging confirmed preferential accumulation of MCC, MCC@TM, and MCC@TM‐αP in tumors following their administration (**Figure** **4**a). In all three groups, the signals peaked at 12 h post‐injection and gradually decreased owing to the metabolism of MCC, MCC@TM, and MCC@TM‐αP. Compared with MCC and MCC@TM, tumors in the MCC@TM‐αP group exhibited the strongest fluorescence signal, attributable to the dual‐targeting effects of tumor‐cell membrane camouflage and PD‐L1 antibody‐mediated binding. At 12 h post‐injection, the tumor signal intensity in the MCC@TM‐αP group was 1.19‐ and 1.38‐fold higher than that in the MCC and MCC@TM groups (Figure 4b). These results confirm the successful enrichment of MCC@TM‐αP at tumor sites through active dual‐targeting functionality.

Biodistribution analysis revealed a significant accumulation of MCC, MCC@TM, and MCC@TM‐αP in the tumor, liver, and spleen, as their fluorescence signals were primarily detected in these organs, whereas minimal signals were observed in the kidney, heart, and lung (Figure S5, Supporting Information). MCC, MCC@TM, and MCC@TM‐αP exhibited similar enrichment profiles. At 12 h post‐administration, quantitative analysis demonstrated significantly higher tumor accumulation of MCC@TM‐αP compared with MCC and MCC@TM, further corroborating the dual‐targeting effect (Figure 4c). All three groups exhibited higher signal intensities in the tumor, liver, and spleen than in other tissues.

Given the efficient MRI capability of MnO_2_, the T_1_‐weighted MRI performance of MCC, MCC@TM, and MCC@TM‐αP was subsequently evaluated. The tumor regions gradually brightened following administration of MCC, MCC@TM, or MCC@TM‐αP, reaching the brightest signal at 8 h (Figure 4d). In contrast, the MR signal after PBS injection did not exhibit time‐dependent changes. At 8 h post‐administration, the signal intensity for MCC@TM‐αP was markedly higher than that of MCC and MCC@TM (Figure 4e), attributable to the enhanced tumor accumulation of MCC@TM‐αP. These findings corroborate the effective T_1_‐weighted MRI performance of the nanoplatforms in tumors.

### 2.4 In Vivo Tumor Therapeutic Efficacy Evaluation

**Figure 5:**
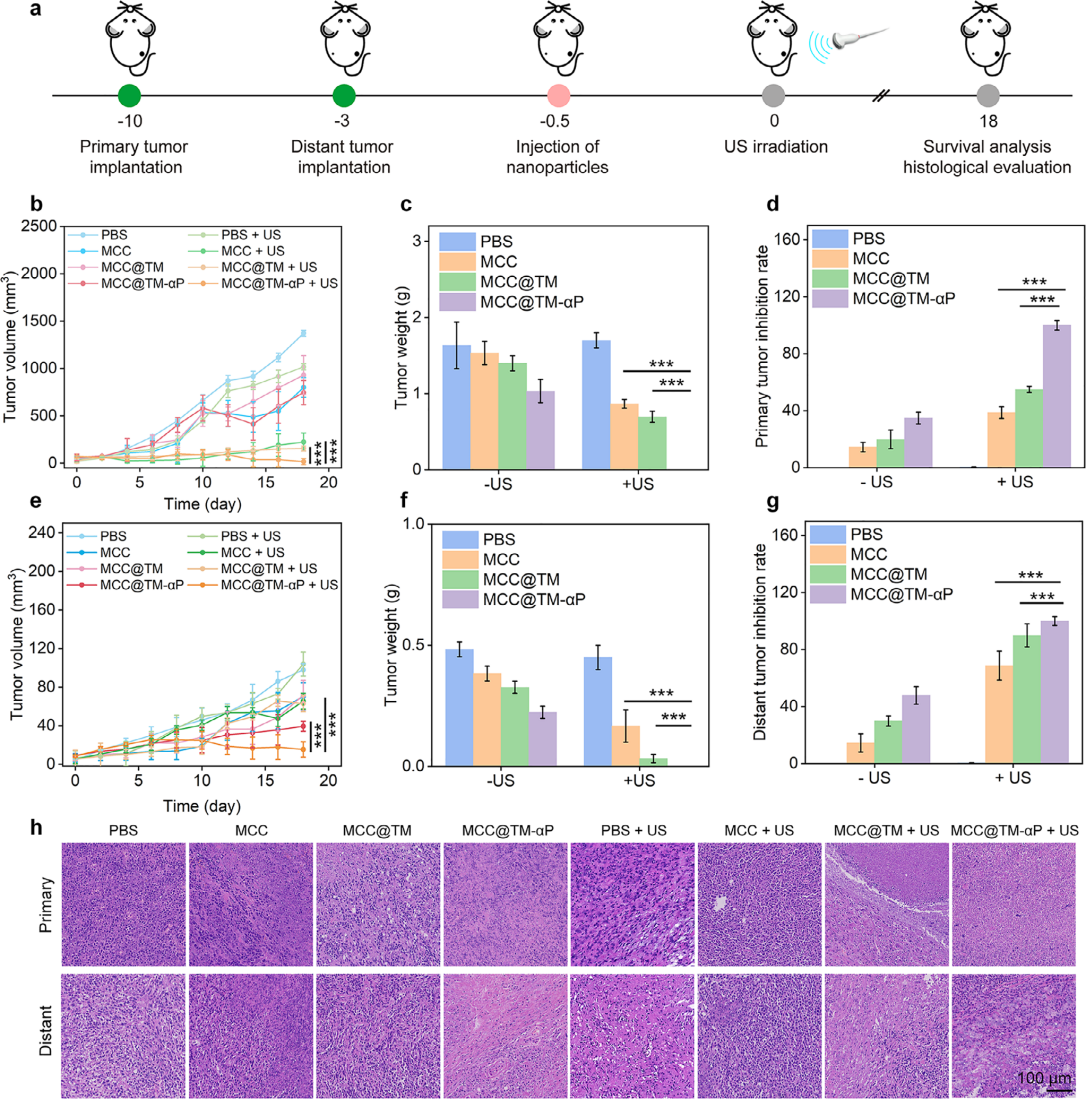
Evaluation of tumor therapeutic efficacy in vivo. a) Schematic of the treatment strategy for 4T1 bilateral tumors. b) Volume of primary 4T1 tumors in mice (*n* = 5). c) Weights of primary 4T1 tumors (*n* = 5). d) Inhibition rates of primary tumors (*n* = 5). e) Volume of distant 4T1 tumors (*n* = 5). f) Weights of distant 4T1 tumors (*n* = 5). g) Inhibition rates of distant tumors (*n* = 5). h) H&E staining analysis of tumors. All data are presented as the mean ± SD (^*^ *p*  < 0.05, ^**^ *p* <0.01, ^***^ *p*  < 0.001; two‐tailed Student's *t*‐tests).

Bilateral 4T1 tumor murine models were used to evaluate the in vivo therapeutic efficacy following tail intravenous injection of MCC, MCC@TM, and MCC@TM‐αP, combined with US exposure of the primary tumors (**Figure** **5**a). Although the growth of primary tumors was suppressed in all treatment groups compared with that in controls, mice in the MCC + US, MCC@TM + US, and MCC@TM‐αP + US groups exhibited substantially lower primary tumor volumes than the other three treatment groups (Figure 5b). Notably, the lowest primary tumor volume was observed in the MCC@TM‐αP + US group after 22 d of treatment. At the end of the treatment period, average tumor weights in the MCC + US (0.86 g), MCC@TM + US (0.69 g), and MCC@TM‐αP + US (0 g) groups were markedly lower than those in the control group (Figure 5c). The primary tumor inhibition rates were 14.4% for MCC, 19.9% for MCC@TM, 34.8% for MCC@TM‐αP, 38.7% for MCC + US, 54.8% for MCC@TM + US, and 100% for MCC@TM‐αP + US (Figure 5d). Following intravenous injection of MCC, MCC@TM, and MCC@TM‐αP combined with US exposure, significant inhibition of distant tumor growth was observed. The MCC + US, MCC@TM + US, and MCC@TM‐αP + US groups demonstrated efficient antitumor effects, with tumor volumes markedly reduced compared with those in the controls at day 22 post‐treatment (Figure 5e). In contrast, MCC, MCC@TM, and MCC@TM‐αP without US exposure showed only moderate inhibition of tumor growth owing to limited antitumor efficacy. The weights of distant tumors were lowest in the MCC@TM‐αP + US group, whereas those in other treatment groups remained higher (Figure 5f). The MCC, MCC@TM, and MCC@TM‐αP groups displayed moderate inhibition rates of 14.4%, 29.8%, and 47.8%, respectively, whereas the MCC + US, MCC@TM + US, and MCC@TM‐αP + US groups reached 68.6%, 89.8%, and 100%, respectively (Figure 5g). The smallest tumor volumes, lowest weights, and highest inhibition rates of distant tumors were consistently observed in the MCC@TM‐αP + US group. Histopathological analysis revealed extensive tumor necrosis in MCC, MCC@TM, MCC@TM‐αP, MCC + US, and MCC@TM + US groups, while control tumors showed minimal necrosis (Figure 5h). Overall, the most pronounced inhibition rates of both primary and distant bilateral tumors were achieved in the MCC@TM‐αP + US group.

Mice in all groups, including the control and treatment groups, maintained stable body weights throughout the study (Figure S6, Supporting Information). No abnormal histological morphology was observed in the organs of treated mice (Figure S7, Supporting Information). These findings confirm the low in vivo toxicity of this therapeutic approach.

### 2.5 In Vivo Anti‐Metastatic Efficacy and Survival Evaluation

**Figure 6:**
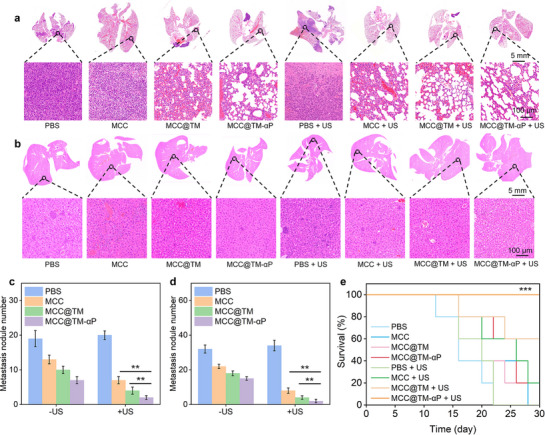
Evaluation of anti‐metastatic efficacy and survival in vivo. a) Analysis of lung metastasis in mice following intravenous injection of MCC, MCC@TM, or MCC@TM‐αP, with or without US treatment. b) Analysis of liver metastasis in mice following intravenous injection of MCC, MCC@TM, or MCC@TM‐αP, with or without US treatment. c) Quantification of lung metastasis nodules (*n* = 5). d) Quantification of liver metastasis nodules (*n* = 5). e) Survival analysis of mice following intravenous injection of MCC, MCC@TM, or MCC@TM‐αP, with or without US treatment (*n* = 5). All data are presented as the mean ± SD (^*^ *p* <0.05, ^**^ *p*  < 0.01, ^***^ *p*  < 0.001; two‐tailed Student's *t*‐tests).

Given the high metastatic potential of 4T1 tumor murine models, the in vivo anti‐metastasis effect was assessed. Compared to controls, all treatment groups exhibited reduced tumor metastatic nodules in the lungs and liver (**Figure** **6**a,b). Notably, the extent of lung and liver metastasis was lower in the MCC + US, MCC@TM + US, and MCC@TM‐αP + US groups than in the MCC, MCC@TM, and MCC@TM‐αP groups. In lung tissues, the number of metastatic nodules was ≈18.9 in controls, compared to 132, 10.1, 7.0, 7.0, 4.3, and 2.5 in the MCC, MCC@TM, MCC@TM‐αP, MCC + US, MCC@TM + US, and MCC@TM‐αP + US groups, respectively (Figure 6c). A notable reduction in liver metastasis was observed in the MCC, MCC@TM, and MCC@TM‐αP groups, regardless of US exposure. with the MCC@TM‐αP + US group exhibiting the lowest metastasis (Figure 6d). The number of liver metastatic nodules was 22.2, 18.3, 15.0, 8.5, 4.0, and 2.0 in the MCC, MCC@TM, MCC@TM‐αP, MCC + US, MCC@TM + US, and MCC@TM‐αP + US groups, respectively. These findings confirm that MCC@TM‐αP + US achieved the most effective anti‐metastasis outcome.

The administration of MCC, MCC@TM, or MCC@TM‐αP combined with US exposure resulted in a marked improvement in the long‐term survival of mice bearing aggressive 4T1 tumors. In the MCC + US and MCC@TM + US groups, 20% and 60% of tumor‐bearing mice, respectively, survived beyond 30 d, compared to 0% survival in the control, MCC, MCC@TM, and MCC@TM‐αP groups (Figure 6e). Notably, all mice in the MCC@TM‐αP + US group maintained 100% survival at the 30‐d endpoint, indicating a durable therapeutic effect. The superior survival in the MCC@TM‐αP + US group can be attributed to its potential efficacy in eradicating tumors and suppressing metastasis.

### 2.6 In Vivo Tumor ICD and Immune Response Analyses

**Figure 7:**
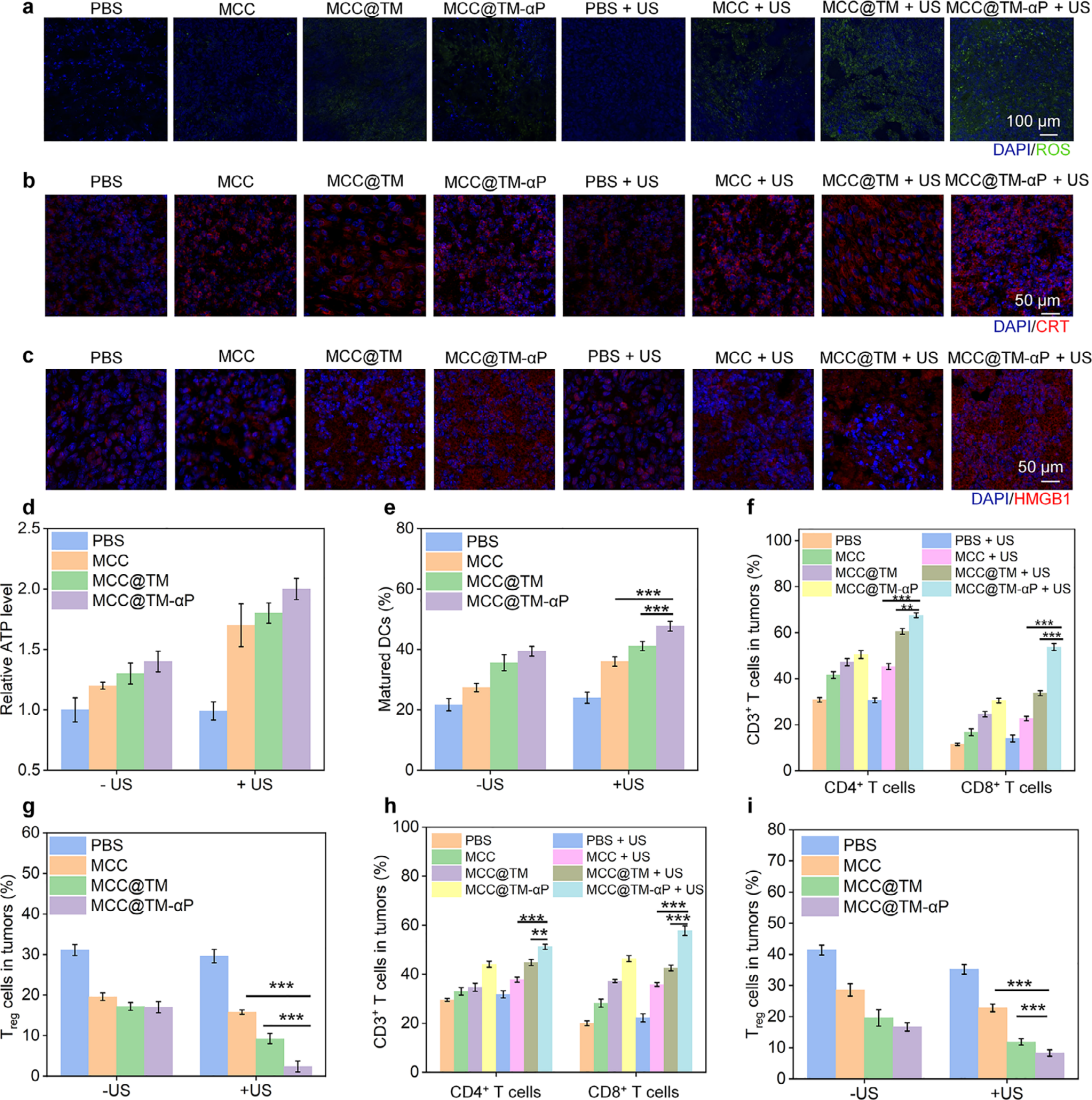
Tumor ICD and immune response analyses. a) ROS production in tumors of mice following intravenous injection of MCC, MCC@TM, and MCC@TM‐αP, with or without US treatment. b) CRT levels in tumors via immunofluorescence staining. c) HMGB1 levels in tumors via immunofluorescence staining. d) ATP levels in tumors (*n* = 3). e) Mature DC levels in various groups (*n* = 3). f) CD4^+^ and CD8^+^ T cell levels in primary tumors (*n* = 3). g) Regulator T cell (T) levels in primary tumors (*n* = 3). h) CD4^+^ and CD8^+^ T cell levels in distant tumors (*n* = 3). i) T_reg_ levels in distant tumors (*n* = 3). All data are presented as the mean ± SD (^*^ *p* <0.05, ^**^ *p*  < 0.01, ^***^ *p*  < 0.001; two‐tailed Student's *t*‐tests).

The production of intratumoral ROS through the combined effects of CDT and SDT was confirmed via fluorescence imaging, which revealed markedly stronger signals in the MCC + US, MCC@TM + US, and MCC@TM‐αP + US groups, whereas weaker signals were observed in the MCC, MCC@TM, and MCC@TM‐αP groups (**Figure** **7**a). ROS generation in the MCC + US, MCC@TM + US, and MCC@TM‐αP + US groups resulted from both CDT and SDT effects, whereas that in the MCC, MCC@TM, and MCC@TM‐αP groups can be attributed solely to the CDT effect. Owing to the dual‐targeting properties of MCC@TM‐αP, the MCC@TM‐αP + US group showed the highest ROS signal intensity (Figure S8, Supporting Information). ROS production‐induced ICD was then evaluated. Compared with the controls, CRT signals in the treated groups were significantly elevated to varying degrees (Figure 7b). Notably, the CRT signal in the MCC@TM‐αP + US group was the strongest, showing a 2.8‐ and 1.6‐fold increase compared with that in the MCC + US and MCC@TM + US groups, respectively (Figure S9, Supporting Information). Elevated levels of HMGB1 were also observed in all treated groups, indicating that ROS‐induced damage promoted HMGB1 secretion from tumor cells (Figure 7c). In particular, analysis revealed that injection of MCC, MCC@TM, or MCC@TM‐αP combined with US exposure triggered significantly greater HMGB1 secretion compared with the corresponding groups without US exposure (Figure S10, Supporting Information). Higher ATP secretion was also detected in tumors following MCC, MCC@TM, or MCC@TM‐αP injection, both without and with US exposure, compared with that in the controls (Figure 7d). Among these groups, the MCC@TM‐αP + US group exhibited the highest ATP levels. These findings confirm that tumor ICD was induced by enhanced oxidative stress.

In vivo immune response activation was assessed, and the effects of ICD were confirmed. Given the induction of ICD in all treated groups, the levels of mature DCs in these groups were markedly elevated compared with those in the control groups (Figure S11, Supporting Information). The MCC@TM‐αP + US group exhibited the highest DC level of 47.7%, representing a 1.3‐ and 1.2‐fold increase compared to that in the MCC + US and MCC@TM + US groups, respectively (Figure 7e). Different immune cell populations within the tumors were analyzed to clarify the in vivo immune activation. In primary tumors, both CD4^+^ and CD8^+^ T cells showed a marked increase following MCC, MCC@TM, and MCC@TM‐αP injection, both with and without US exposure (Figures S12 and S13, Supporting Information). The levels of CD4^+^ T cells in the MCC + US, MCC@TM + US, and MCC@TM‐αP + US groups reached 45.3%, 60.6%, and 67.6%, respectively, among all CD3^+^ T cells (Figure 7f). Notably, primary tumors exhibited the highest level of CD8^+^ T cells, with a value at least 1.59‐fold higher than that in the other groups (Figure 7f). Concurrently, the proportion of T_reg_ cells decreased following MCC, MCC@TM, and MCC@TM‐αP injection, both with and without US exposure, with the lowest level (2.4%) observed in the MCC@TM‐αP + US group (Figure 7g; Figure S14, Supporting Information). Analysis of immune cells in distant tumors further demonstrated the induction of a systemic immune response contributing to tumor inhibition. This effect can be attributed to US‐activated nanoparticles generating ROS, which induced ICD and promoted the release of tumor antigens and danger signals. These signals initiated DC maturation, and the activated DCs subsequently migrated to lymph nodes to present antigens to CD8^+^ T cells. This cascade triggered a systemic antitumor immune response, leading to the substantial expansion and activation of cytotoxic T lymphocytes (CTLs). ​​These activated CTLs then homed to and infiltrated distant metastatic sites, such as the lungs and liver, where they directly mediated tumor cell killing. Furthermore, the robust immune response, characterized by elevated levels of effector cytokines such as interferon‐γ secreted by CTLs and other immune cells, contributed to remodeling the immunosuppressive TME, potentially impairing the function and stability of regulatory T cells (T_regs_). Collectively, these mechanisms significantly inhibited metastatic progression. MCC, MCC@TM, and MCC@TM‐αP injection combined with US exposure markedly increased the CD4^+^ and CD8^+^ T cell populations (Figures S15 and S16, Supporting Information). Both CD4^+^ and CD8^+^ T cells exhibited the highest levels among all CD3^+^ T cells in distant tumors for the MCC@TM‐αP + US group (Figure 7h). Moreover, MCC, MCC@TM, and MCC@TM‐αP injection, both without and with US exposure, reduced T_reg_ levels in distant tumors (Figure S17, Supporting Information). Distant tumors in the MCC@TM‐αP + US group exhibited a T_reg_ level of only 8.6% (Figure 7i). These findings clearly demonstrate the activation of an in vivo immune response, contributing to the inhibition of tumor growth and metastasis.

## 3 Conclusion

In summary, we have reported a modular nanoplatform (MCC@TM‐αP) for *reprogramming* the TME through *sequential* cascade actions to enhance breast cancer immunotherapy. MCC@TM‐αP consisted of a hybrid nanoparticle composed of MnO_2_, CaO_2_, and Ce6, camouflaged with a 4T1 tumor cell membrane and conjugated with a PD‐L1 antibody (αP) via a GSH‐responsive fragment. This study demonstrated that the MCC@TM‐αP nanoplatform effectively addressed the tripartite challenges of the breast cancer TME through sequential and stimuli‐responsive actions. By synergistically combining hypoxia alleviation, GSH depletion, and enhanced SDT efficacy, the system not only achieved direct tumor ablation but also dynamically reprogrammed the immunosuppressive TME. The GSH‐responsive release of αP further leveraged this remodeled TME to activate systemic antitumor immunity, as evidenced by increased CD8^+^ T cell infiltration and reduced metastasis in murine models. The integration of biomimetic targeting (via cell membrane camouflage), microenvironment‐responsive drug release, and multimodal therapy provides a blueprint for developing next‐generation nanoplatforms to overcome therapy‐resistant cancers. Despite limitations such as model differences and insufficient long‐term toxicity evaluation, the modular design demonstrates translational potential across cancer types. Future work should focus on large‐animal and clinical translation. Overall, this study mechanistically demonstrates that stepwise disruption of both physical and immune barriers within the TME significantly enhances immune cell infiltration and activation, thereby maximizing the potential of cancer immunotherapy.

## 4 Experimental Section

### Synthesis of MCC

A 1.3 m CaCl_2_ solution was added to 60 mL of anhydrous methanol at room temperature (25 °C) under continuous vigorous stirring for 10 min. Then, 2 mL of 3% H_2_O_2_ solution and 0.6 mL of ammonia solution were slowly added to the mixture. After the reaction and subsequent centrifugation, CaO_2_ nanoparticles were obtained. KMnO_4_ (32 mg) and BSA (250 mg) were reacted for 3 h, followed by purification through ultrafiltration to yield the BSA–MnO_2_ nanoparticles. Then, 50 µL of Ce6 in THF (500 µg mL^−1^) and 50 µL of CaO_2_ aqueous solution were added to 1 mL of BSA–MnO_2_ solution, followed by sonication for 30 min. The mixture was shaken for 12 h to evaporate THF, yielding MCC nanoparticles.

### Synthesis of MCC@TM

Approximately 9 × 10^6^ 4T1 cancer cells were resuspended in 1 mL of PBS (cell suspension concentration: 9 × 10^6^ cells mL^−1^) and then treated using a SCIENTZ‐IID ultrasonic cell disruptor (Ningbo Xinzhi Biotechnology Co., Ltd.) equipped with a 6 mm‐diameter titanium alloy probe (250 W power, in an ice bath) for 30 min to obtain cell membrane fragments. Subsequently, MCC (1 mL, 200 µg mL^−1^) was mixed with 1 mL of the membrane solution and extruded through a lipid extruder to form MCC@TM, followed by purification.

### Synthesis of MCC@TM‐αP

A solution containing 5 µL of a 1 m stock solution of cystamine dihydrochloride in PBS, 0.02 mmol EDC, and 0.02 mmol NHS was added to MCC@TM solution (100 µg mL^−1^), and the mixture was incubated at room temperature in the dark for 1 h. Subsequently, PD‐L1 antibody (1 µL, 8.29 mg mL^−1^) was added, and the reaction was continued for 24 h to achieve surface conjugation. The final product, MCC@TM‐αP, was transferred to a Millipore Amicon Ultra‐4 ultrafiltration centrifuge tube (molecular weight cutoff 50 kDa) and centrifuged at 4 °C and 500 × *g*. During centrifugation, multiple washes with PBS were performed to remove unreacted small‐molecule reagents and antibodies. Centrifugation was terminated when the concentrated volume in the ultrafiltration tube decreased to ≈200 µL, yielding the purified MCC@TM‐αP concentrate.

### In Vitro MRI Properties

MCC, MCC@TM, and MCC@TM‐αP solutions (25 µg mL^−1^ each) were prepared. T_1_‐weighted MR images of these solutions were acquired using a clinical 3.0 T MRI system (MAGNETOM Vida, Siemens Healthineers, Germany). Imaging was performed with a fast spin‐echo technique. Key parameters were set as follows: repetition time = 500 ms, echo time = 20 ms, slice thickness = 3.0 mm, matrix size = 256 × 256, and field of view = 200 mm × 200 mm. All samples were imaged at room temperature using the same scanning sequence to ensure consistency of imaging parameters.

### Singlet Oxygen (^1^O_2_) Generation Assay

Quantitative analysis of ^1^O_2_ detection was performed using the fluorescence probe Singlet Oxygen Sensor Green (SOSG; Thermo Fisher Scientific; S36002). The SOSG stock solution was prepared by dissolving 100 µg of SOSG powder in 330 µL of methanol, yielding a stock solution of ≈500 µm. Then, 3 µL of this stock solution was added to 25 µg mL^−1^ solutions of MCC, MCC@TM, and MCC@TM‐αP to achieve a final concentration of ≈5 µm in the reaction system. Subsequently, the samples were irradiated with US (1.0 MHz, 1.0 W cm^−2^) for 2–10 min, and ^1^O_2_ generation efficiency was quantified by measuring changes in SOSG fluorescence intensity.

### Hydroxyl Radical (·OH) Generation Assay

The ·OH production was assessed by incubating 3 mL of MCC, MCC@TM, and MCC@TM‐αP (25 µg mL^−1^) in TMB solution containing H_2_O_2_ (100 µm) and GSH (100 µm). Changes in TMB absorbance were measured using UV‐Vis spectrophotometry.

### αP Release Assay

MCC@TM‐αP solutions (25 µg mL^−1^) were treated with GSH (10 µm) for 12 h. The solutions were then collected via ultrafiltration, and the αP content was measured using a BCA protein assay kit. Relative release percentages of αP were subsequently calculated.

### Cell Endocytosis Assessment

1.5 × 10^5^ 4T1 cells were seeded into a 6‐well plate containing RPMI‐1640 medium supplemented with 10% fetal bovine serum (FBS) and 1% penicillin–streptomycin. The cells were incubated at 37 °C in a 5% CO_2_ incubator until 70–80% confluence was reached. Fresh complete medium containing 25 µg mL^−1^ MCC, MCC@TM, or MCC@TM‐αP nanoparticles was then added. After 24 h of incubation, the cells were washed with PBS to remove free particles. Confocal laser scanning microscopy (CLSM) was used to analyze cellular uptake.

### Cell Therapeutic Effect Assessment

1.0 × 10^4^ 4T1 cells were cultured in RPMI‐1640 medium supplemented with 10% fetal bovine serum (FBS) and 1% penicillin–streptomycin containing MCC, MCC@TM, or MCC@TM‐αP (25 µg mL^−1^ each) for 24 h with US treatment (equipment: DJO 2776 portable ultrasound therapy device, DJO, LLC, USA, with a 2 cm‐diameter treatment head; parameters: 1.0 W cm^−2^, 1.0 MHz, 3 min duration). Cell therapeutic efficacy was determined by analyzing cell viability using the CCK‐8 assay.

### Intracellular ROS Assessment

After incubation with MCC, MCC@TM, or MCC@TM‐αP (25 µg mL^−1^ each) for 24 h, 4T1 cancer cells (2.5 × 10^5^) were further co‐incubated with the ROS probe H_2_DCFDA for 30 min. The cells were then subjected to US treatment (equipment: DJO 2776 portable ultrasound therapy device, with a 2 cm diameter treatment head; parameters: 1.0 W cm^−2^, 1.0 MHz, 3 min duration). ROS signals were visualized using CLSM (equipment: Zeiss LSM 700; parameters: excitation wavelength 488 nm, bandpass filter 525/50 nm). Mean fluorescence intensity (MFI) of cells from three independent fields was analyzed using ImageJ software. MFI values were normalized to those of the untreated control group (PBS, no US) and expressed as relative fluorescence intensity.

### Intracellular GSH Assessment

After incubation with MCC, MCC@TM, and MCC@TM‐αP (25 µg mL^−1^), 4T1 cells were treated with US (1.0 MHz, 1.0 W cm^−2^, and 3 min) and subsequently collected. GSH levels in different groups were analyzed using a GSH and GSSG detection kit (S0053; Beyotime Biotechnology Co., Ltd.) according to the manufacturer's instructions.

### In Vitro ICD Assessment

Following the above treatments, extracellular solutions were collected to determine ATP and HMGB1 contents. The 4T1 cells were collected and stained with DAPI and CRT antibodies for assessment of CRT exposure levels.

### Animal Models

All experimental procedures were approved by the Donghua University Animal Care and Use Committee. BALB/c mice (4–5 weeks old) were inoculated in the right and left flanks with 4T1 cells (3 × 10^6^ cells in 50 µL PBS) at a 7‐day interval to establish tumor models.

### Tumor Enrichment and Biodistribution Assessment

4T1 tumor‐bearing mice received intravenous injections of MCC, MCC@TM, or MCC@TM‐αP (300 µg mL^−1^, 200 µL each) in PBS. Real‐time fluorescence images were captured to determine the time of maximum tumor enrichment (T_max_). Following euthanasia, organs and tumors were collected, and fluorescence imaging of these tissues was performed to assess biodistribution by measuring signal intensity.

### Tumor MRI

4T1 tumor‐bearing mice received intravenous injections of MCC, MCC@TM, or MCC@TM‐αP (300 µg mL^−1^, 200 µL each) in PBS. A T_1_‐weighted MRI was conducted, and the resulting images were obtained and analyzed.

### Therapeutic Performance Assessment

4T1 tumor‐bearing mice received intravenous injections of PBS (PBS, PBS + US groups), MCC (MCC, MCC + US groups), MCC@TM (MCC@TM, MCC@TM + US groups), or MCC@TM‐αP (MCC@TM‐αP, MCC@TM‐αP + US groups). At 24 h post‐injection, US treatment was applied to primary tumors in the PBS + US, MCC + US, MCC@TM + US, and MCC@TM‐αP + US groups. Tumor dimensions were recorded using the formula: volume = (length × width^2^)/2. Tumor photographs, tumor weights, animal body weights, survival, and H&E staining were obtained.

### Anti‐Metastasis Performance Assessment

4T1 tumor‐bearing mice underwent the previously described treatment procedures. Following euthanasia, the liver and lungs were collected, processed for histological staining, and metastatic tumor nodules in the lungs and liver were counted.

### Intratumoral ROS Assessment

4T1 tumor‐bearing mice received intravenous injections of MCC, MCC@TM, MCC@TM‐αP, or PBS, followed by local injection of the ROS probe H_2_DCFDA into tumors. After US treatment, tumors were collected, sectioned, and analyzed using CLSM. Fluorescence images were obtained and quantified to assess intratumoral ROS levels.

### Tumor ICD Marker Assessment

4T1 tumor‐bearing mice received intravenous injections of MCC, MCC@TM, MCC@TM‐αP, and PBS, followed by US treatment of primary tumors. Tumors were collected after euthanasia, minced into small pieces (≈1–2 mm^3^), and mechanically homogenized at 4 °C using a tissue homogenizer. The homogenate was filtered through a 70 µm cell strainer to remove undissociated tissue fragments. The filtrate was centrifuged at 3000 × *g* for 15 min at 4 °C, and the supernatant was collected as the tumor tissue fraction. ATP content was quantified using the Beyotime ATP Assay Kit (S0026). Tumors were also sectioned and stained with DAPI and CRT/HMGB1 antibodies, and the stained sections were analyzed using CLSM.

### In Vivo Immune Response Assessment

Following treatment procedures for 7 and 14 d, tissue samples were collected after euthanasia. Samples were digested and processed into single‐cell suspensions using Ficoll density gradient centrifugation. Various immune cell populations were isolated from the suspensions and stained with fluorophore‐conjugated antibodies: BV605‐CD45, APC‐CD3, FITC‐CD4, PE‐CD8b, Alexa Fluor 647‐Foxp3, PE‐CD25, FITC‐CD11c, APC‐CD80, and PE‐CD86 (all antibodies purchased from BD Biosciences, USA). Flow cytometric analysis was performed using a full‐spectrum analysis flow cytometer (FACSMelody), with at least 10^5^ cells collected per sample. Data analysis was conducted using FlowJo software (v10.8), applying gating strategies based on forward scatter/side scatter plots and specific antibody expression to identify T and dendritic cell subsets.

### Statistical Analysis

No data pre‐processing was performed in this study. Data were presented as mean ± SD), and the sample size (n) was indicated for each analysis. Significant differences were evaluated using two‐tailed Student's *t*‐tests. Levels of significance were denoted as ^*^ *p* <0.05, ^**^ *p* <0.01, and ^***^ *p* <0.001. Statistical analyses were conducted using GraphPad Prism (v9.0).

### Humanitarian Endpoint Indicators

Humane endpoints included tumor volume exceeding 1500 mm^3^, body weight loss greater than 20%, severe activity limitation, or the onset of significant pain. When any of these criteria were met, euthanasia was performed immediately to minimize animal suffering.

### Ethics Statement

All animal experiments were conducted in strict accordance with the Animal Research: Reporting of In Vivo Experiments (ARRIVE) guidelines and adhered to the ethical principles approved by the Animal Protection and Use Committee of Donghua University (approval No.: DHUEC‐NSFC‐2022‐16).

## Conflict of Interest

The authors declare no conflicts of interest.

## Supporting information



Supporting Information

## Data Availability

The data that support the findings of this study are available from the corresponding author upon reasonable request.
