# Supplementary material for: Sequential Tumor Microenvironment Reprogramming by Nanoplatform Potentiates Sonodynamic‐Chemodynamic Therapy and Immune Checkpoint Blockade in Breast Cancer
Source: Adv Sci (Weinh). 2025 Oct 24;13(1):e12135. doi: 10.1002/advs.202512135 (PMC12767116; doi:10.1002/advs.202512135)
Supplement: Supplementary file 1 — Supporting Information [file ADVS-13-e12135-s001.docx]

Copyright WILEY-VCH Verlag GmbH & Co. KGaA, 69469 Weinheim, Germany, 2018.

Supporting Information

Sequential Tumor Microenvironment Reprogramming by Nanoplatform Potentiates Sonodynamic-Chemodynamic Therapy and Immune Checkpoint Blockade in Breast Cancer

*Yang Yu^#^, Zheming Song^#^, Anni Zhu, Jingchao Li^*^, Rujia Fan^*^, Bing Xiao^*^*

Dr. Y. Yu

Department of Breast Surgery, Henan Provincial People’s Hospital, Zhengzhou University People’s Hospital, Henan University People’s Hospital, Zhengzhou 450003, Henan, China

Z. Song, A. Zhu, Prof. J. Li

State Key Laboratory of Advanced Fiber Materials, College of Biological Science and Medical Engineering, Donghua University, Shanghai 201620, China

E-mail: jcli@dhu.edu.cn

Prof. R. Fan

Department of Obstetrics and Gynecology, Henan Provincial People’s Hospital, Zhengzhou University People’s Hospital, Henan University People’s Hospital, Zhengzhou 450003, Henan, China

E-mail: fanrujia1980@126.com

Dr. B. Xiao

Department of Radiology, Sir Run Run Shaw Hospital, Zhejiang University School of Medicine, Hangzhou 310016, China

E-mail: xiaobingphd@zju.edu.cn

# 1. Supporting Figures


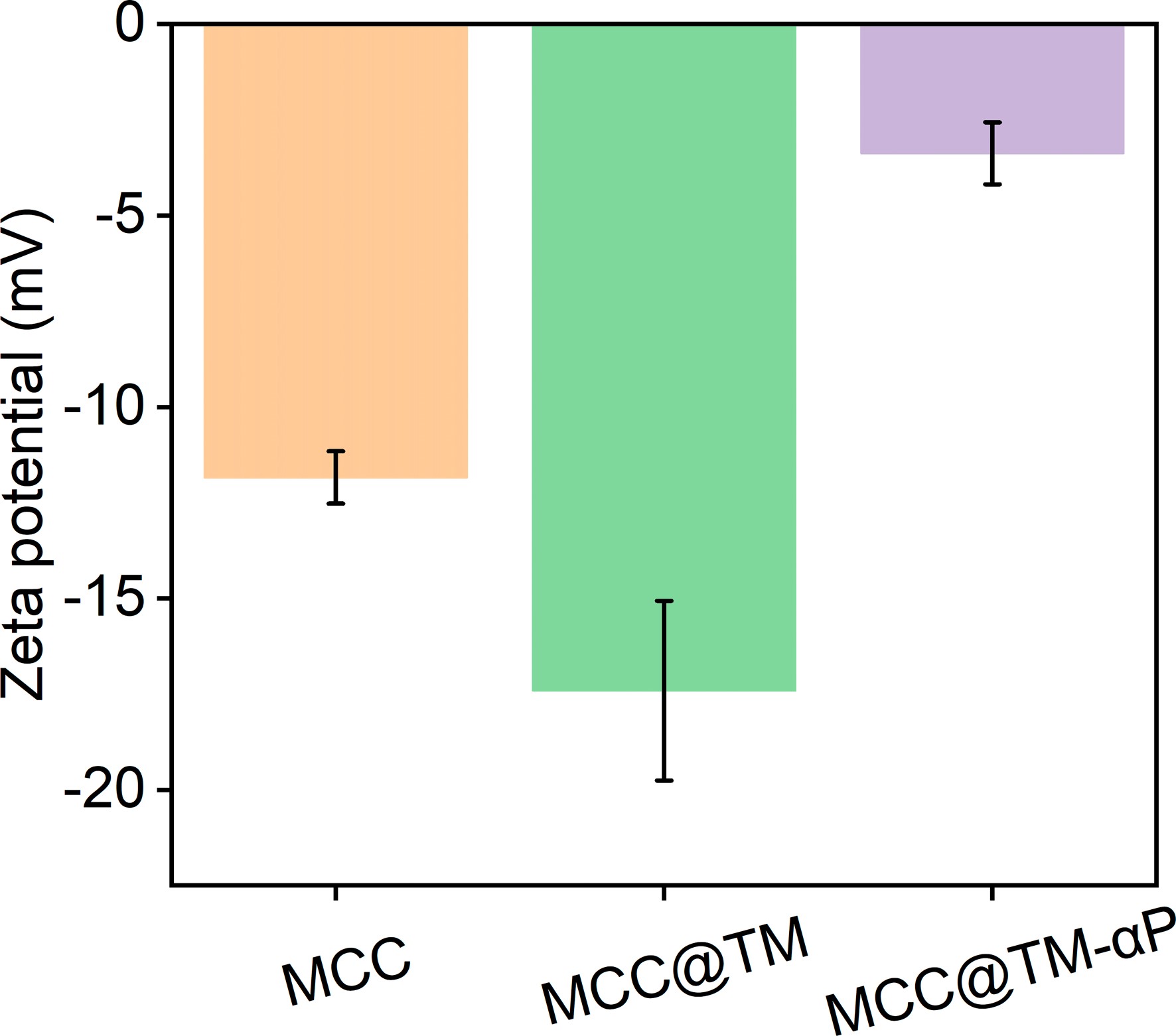


**Figure S1.** Zeta potentials of MCC, MCC@TM and MCC@TM-αP (n = 3).


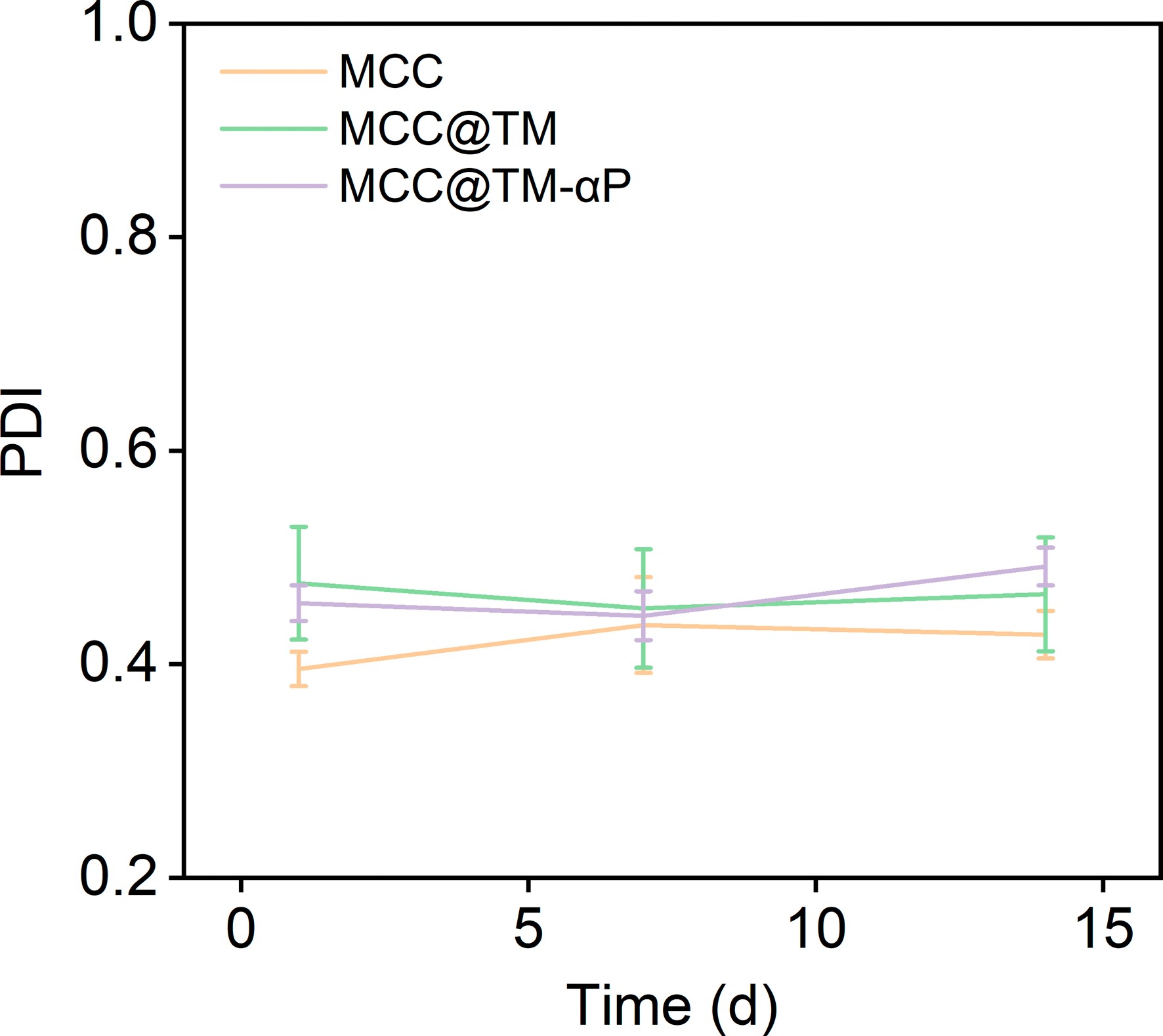


**Figure S2.** Hydrodynamic diameter analysis of MCC, MCC@TM and MCC@TM-αP for a total 14 days (n = 3).


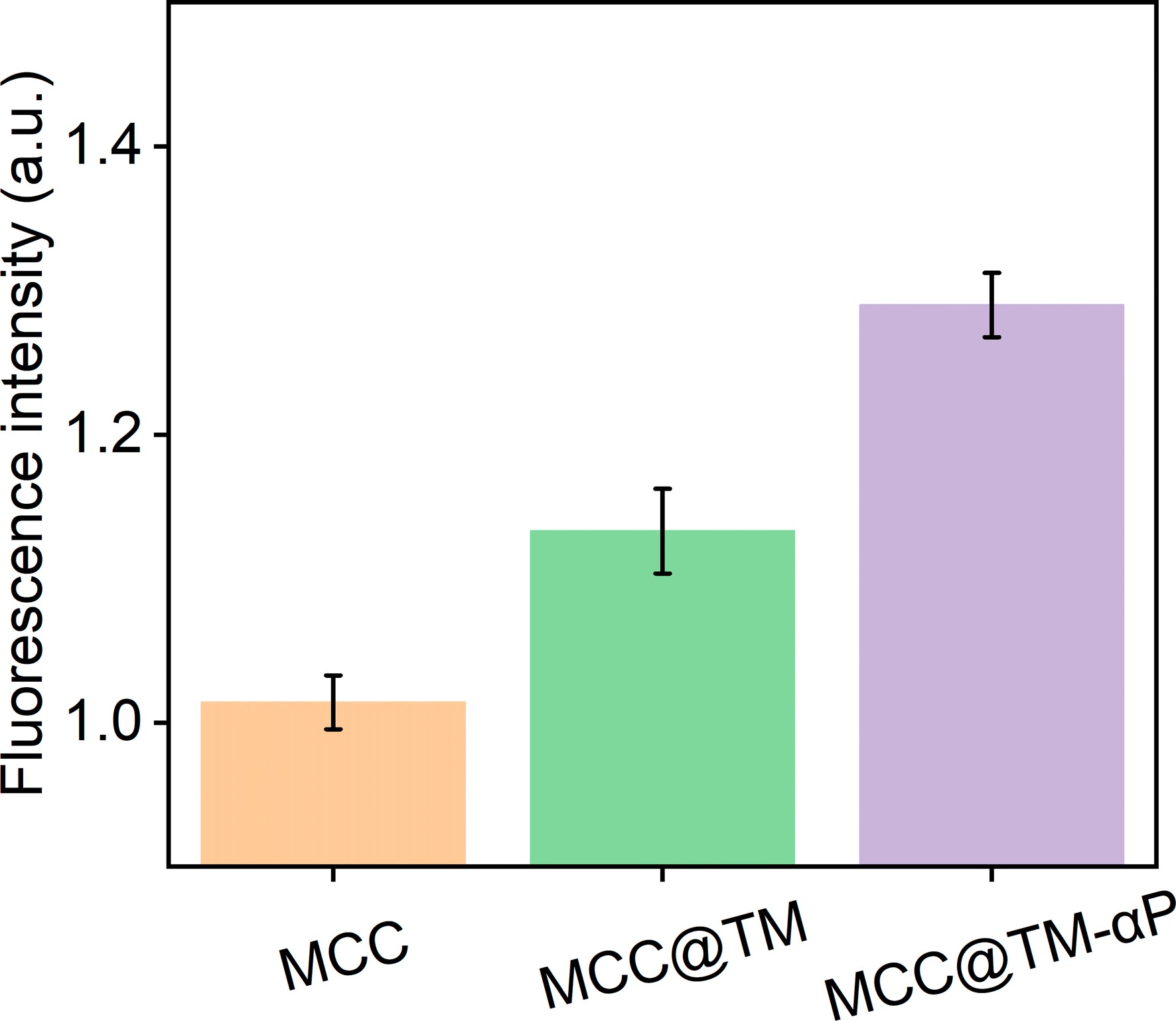


**Figure S3.** Cellular uptake analysis of MCC, MCC@TM and MCC@TM-αP after incubation with 4T1 cells (n = 3).


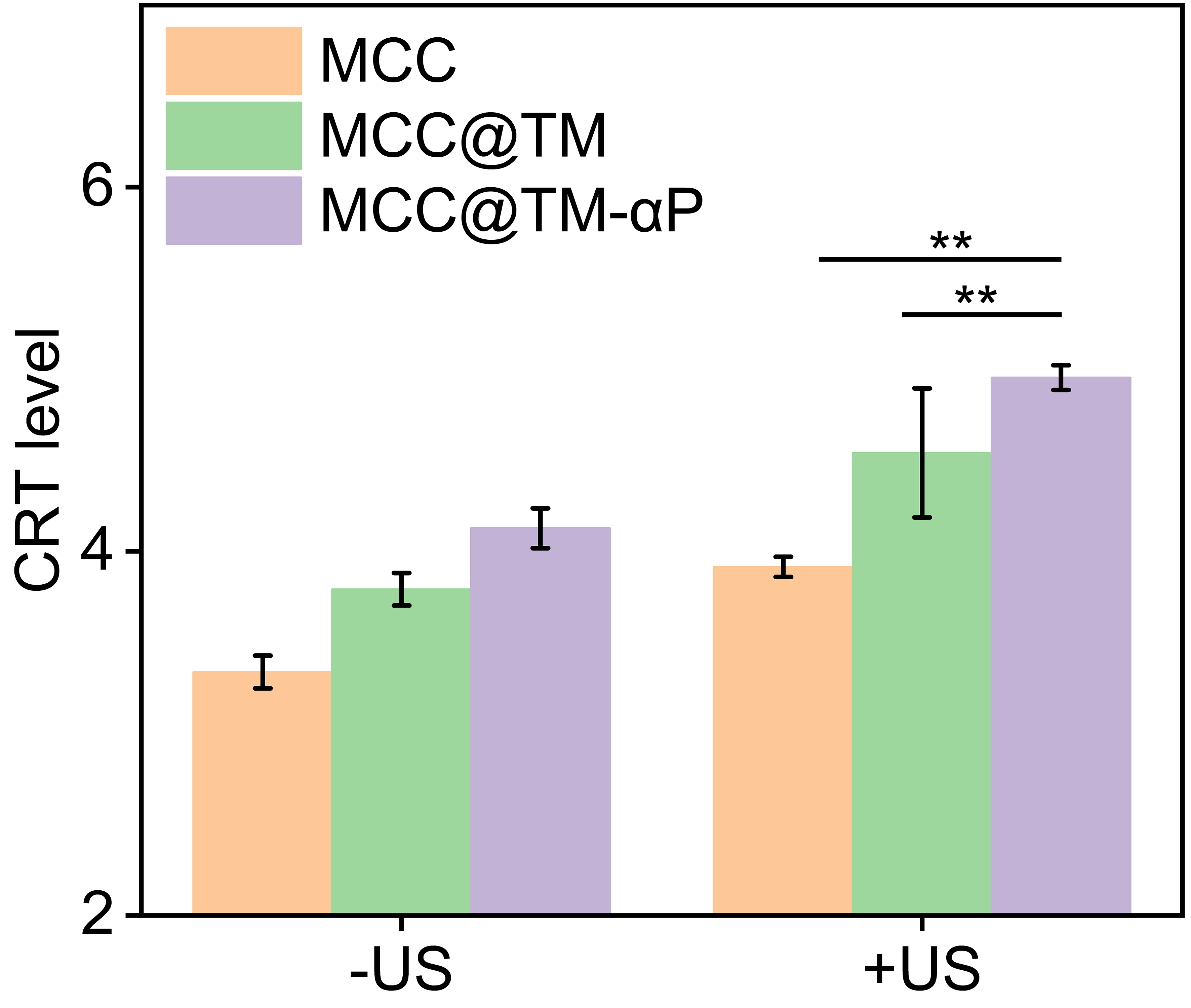


**Figure S4.** CRT signal analysis in 4T1 cells after incubations with MCC, MCC@TM and MCC@TM-αP with US irradiation (n = 3). All data are presented as mean ± SD (**p < 0.01, two-tailed Student’s t-tests).


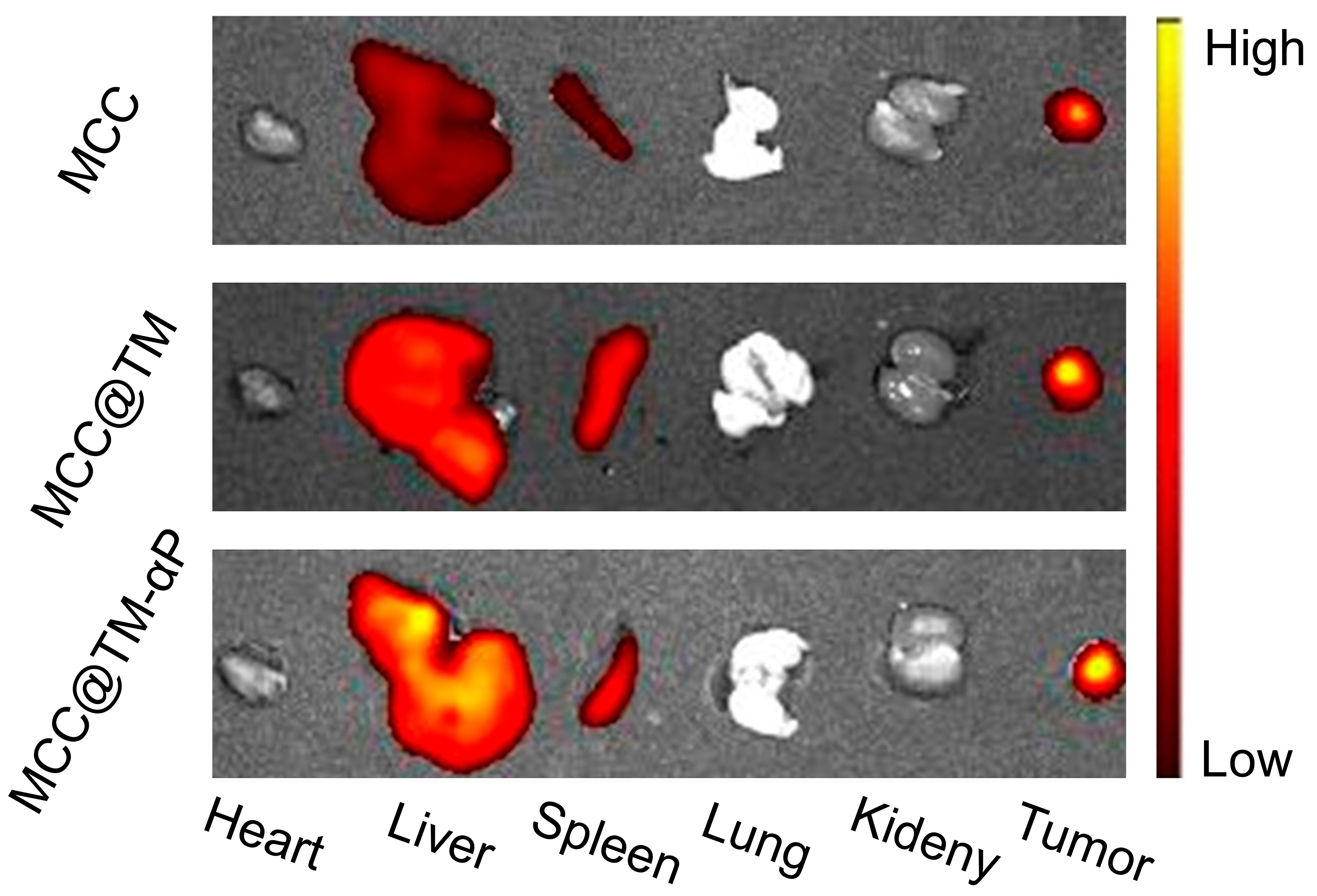


**Figure S5.** Biodistribution analysis of MCC, MCC@TM and MCC@TM-αP in 4T1 tumor-bearing mice after tail intravenous injection (n = 3).


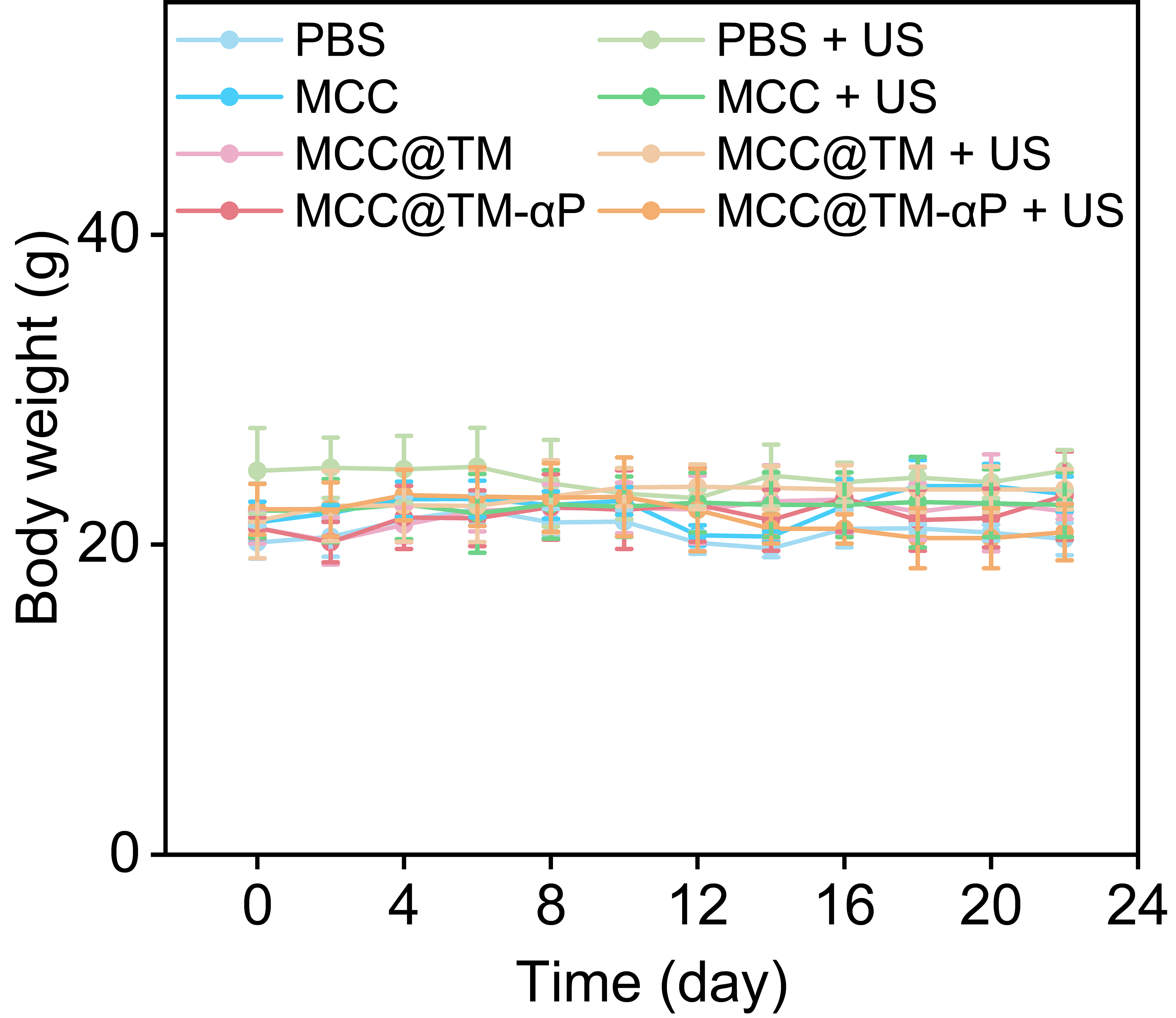


**Figure S6.** The body weights of 4T1 tumor-bearing mice in different treatment groups (n = 5).


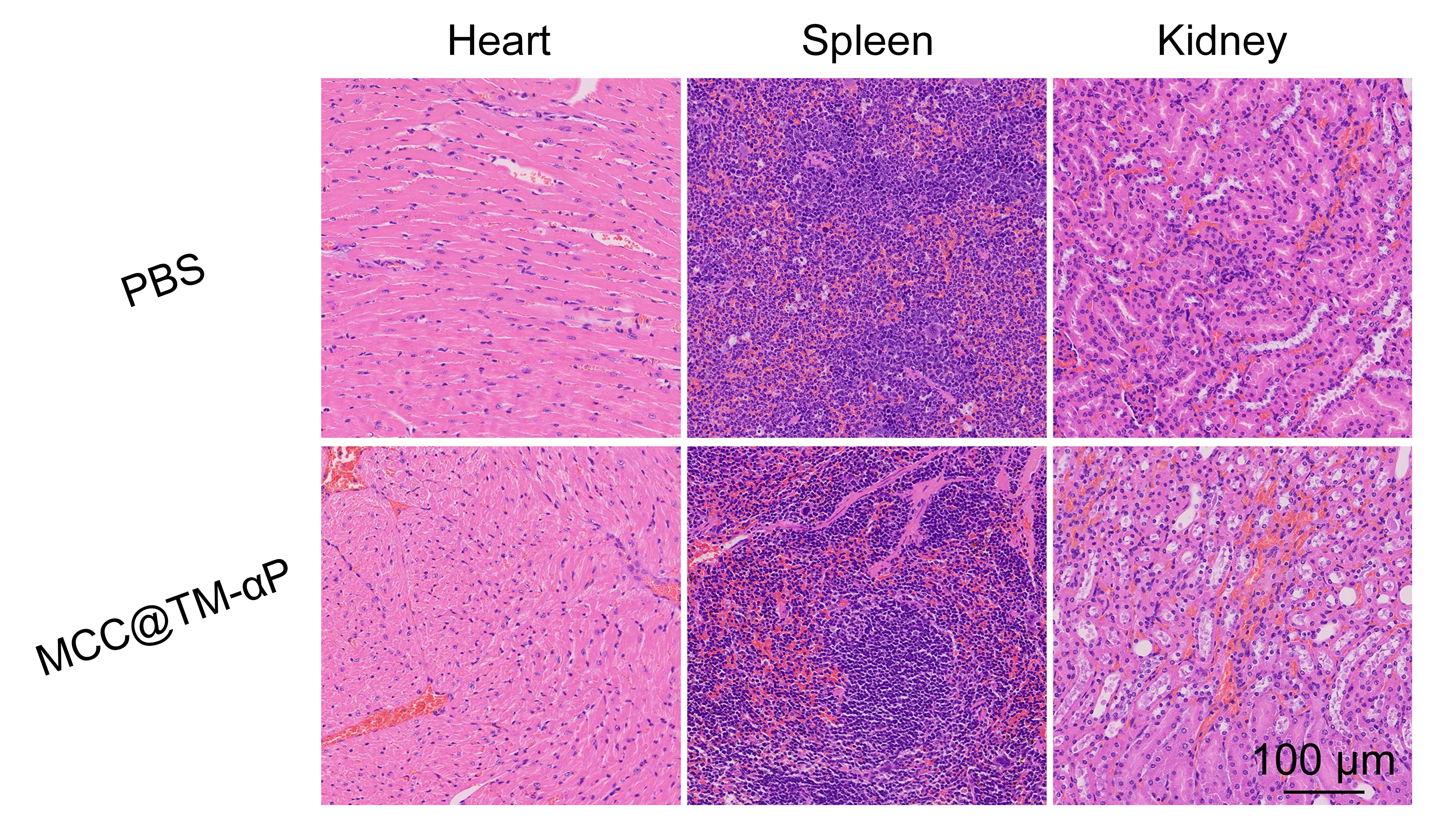


**Figure S7.** H&E staining images of heart, spleen and kidney in different treatment groups.

**
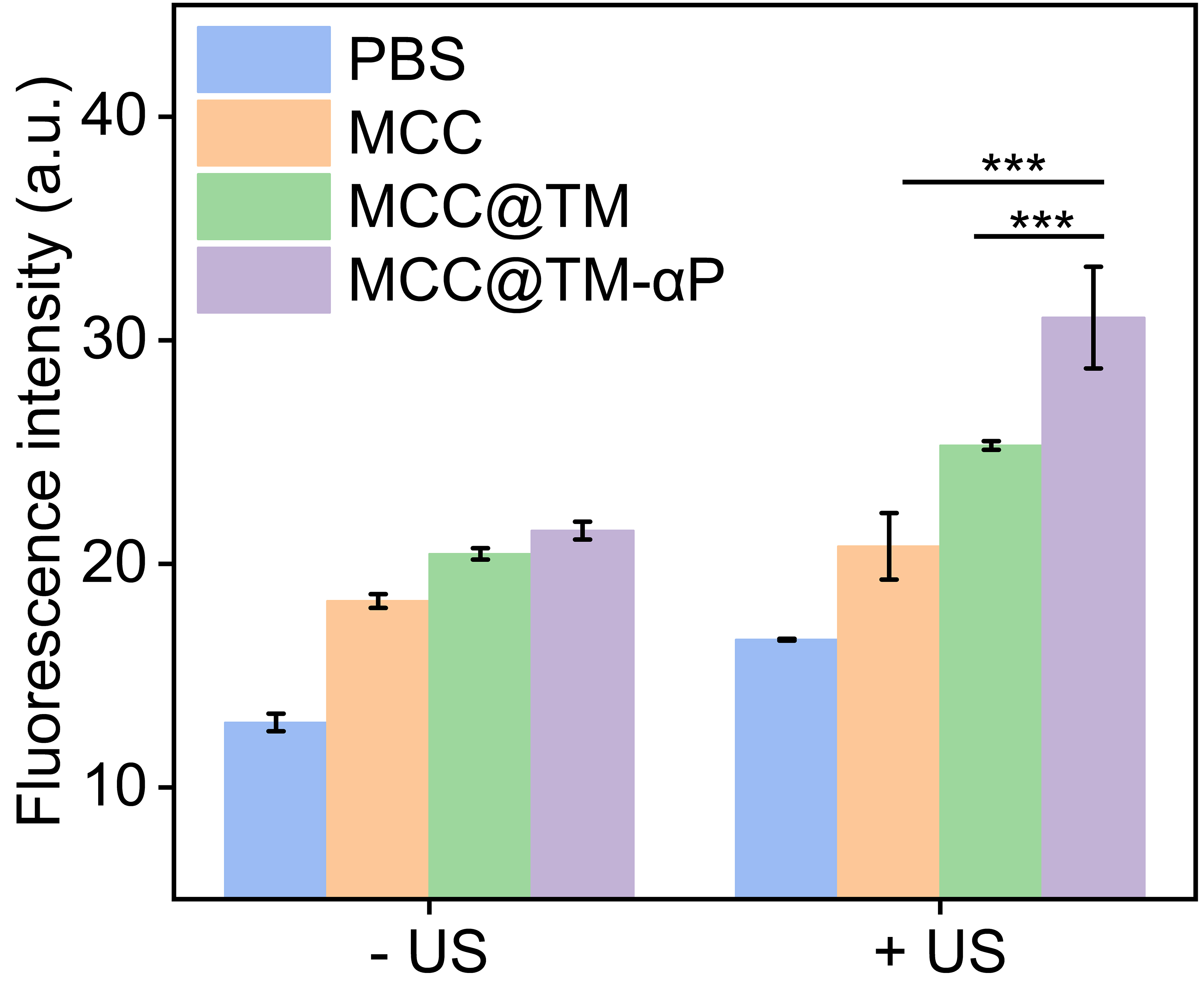
**

**Figure S8.** Analysis of ROS levels in tumors of 4T1 tumor-bearing mice in different treatment groups (n = 3). All data are presented as mean ± SD (***p< 0.001, two-tailed Student’s t-tests).


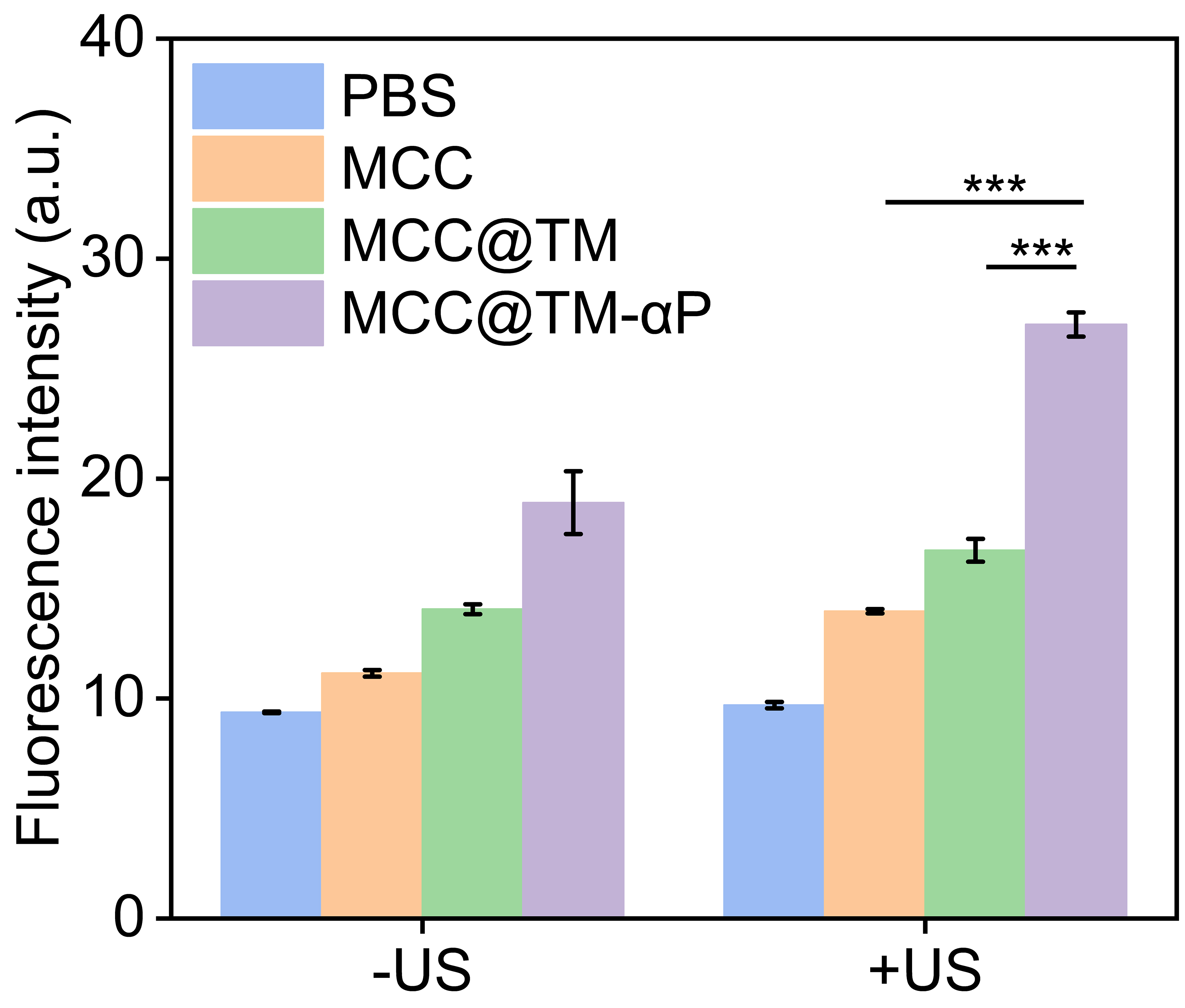


**Figure S9.** CRT staining fluorescence intensity of tumors from various treated mice (n = 3). All data are presented as mean ± SD (***p< 0.001, two-tailed Student’s t-tests).


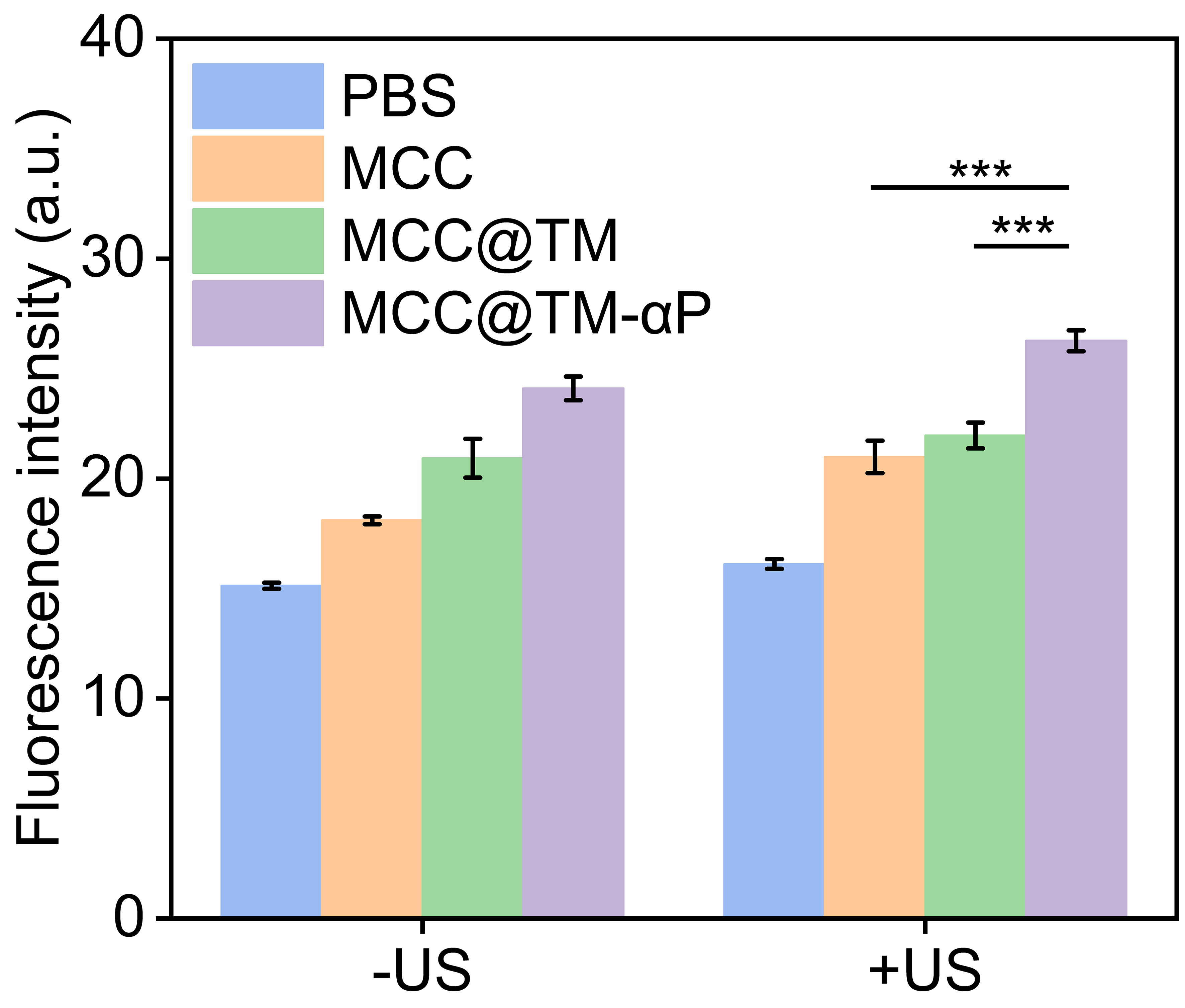


**Figure S10.** HMGB1 staining fluorescence intensity of tumors from various treated mice (n = 3). All data are presented as mean ± SD (***P< 0.001, two-tailed Student’s t-tests).


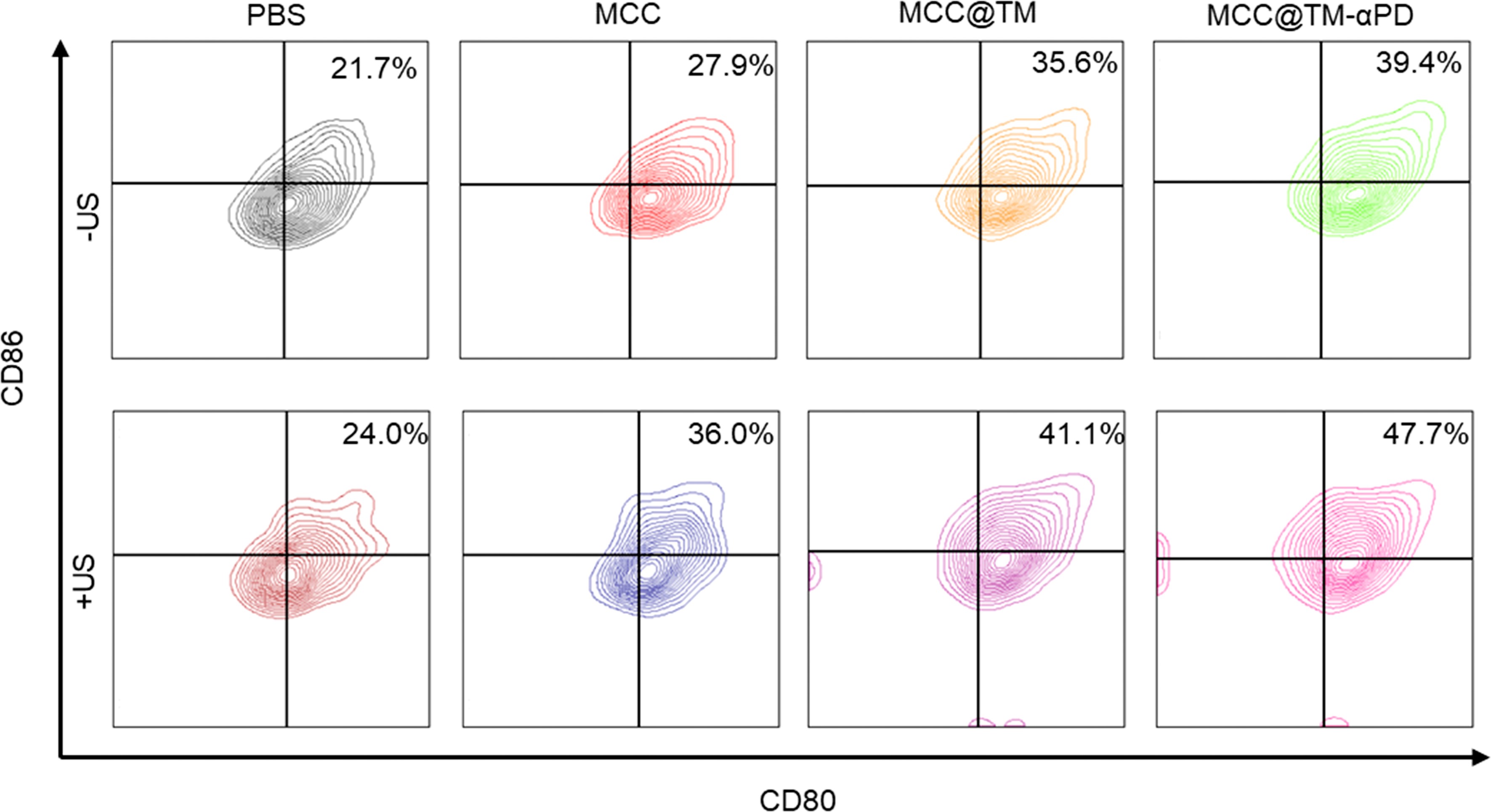


**Figure S11.** Flow cytometry assay of matured DCs (CD80^+^CD86^+^) in tumor draining lymph nodes in various treatment groups.


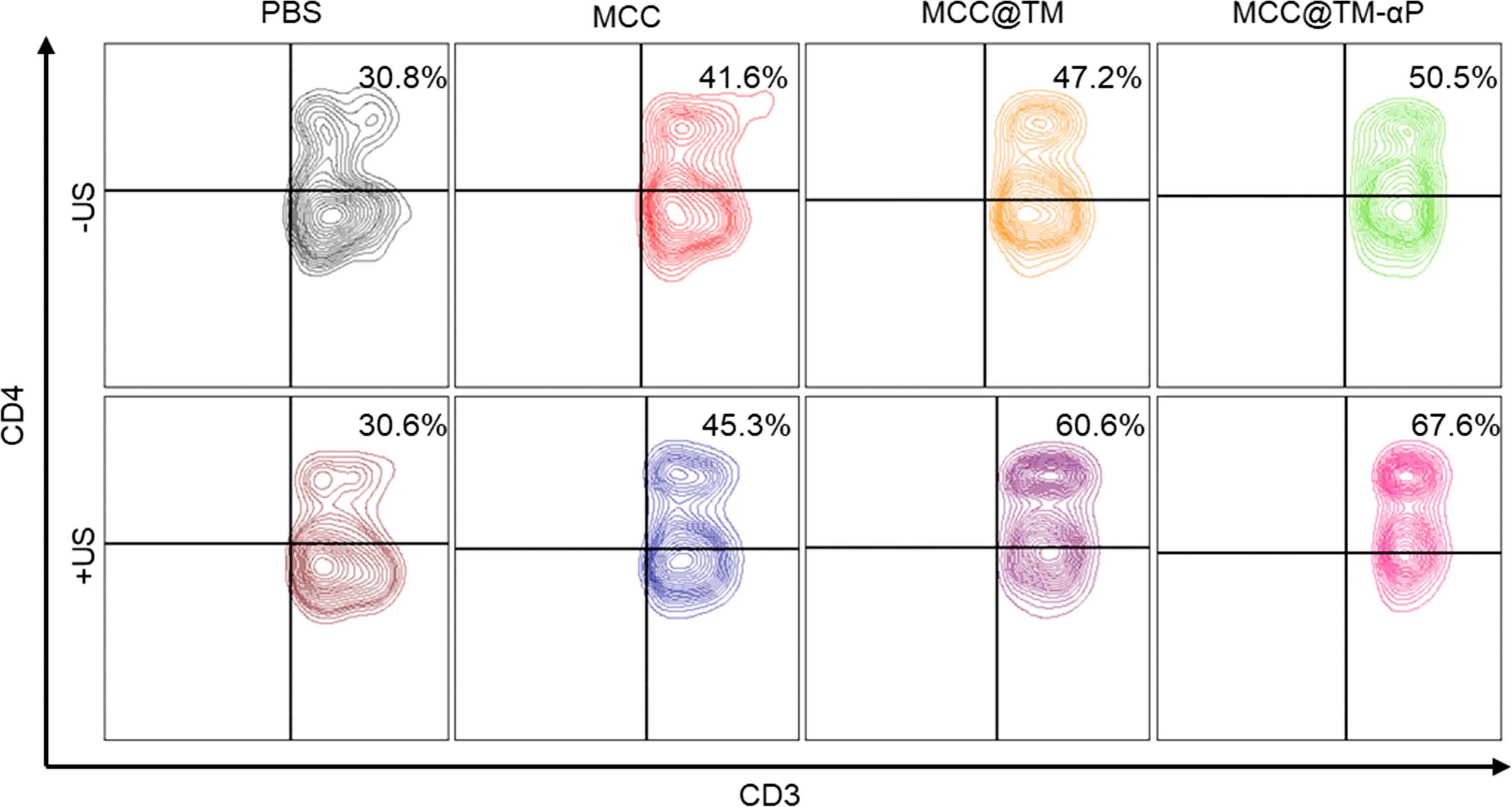


**Figure S12.** Flow cytometry assay of CD3^+^CD4^+^ T cells in primary tumors of mice in various treatment groups.


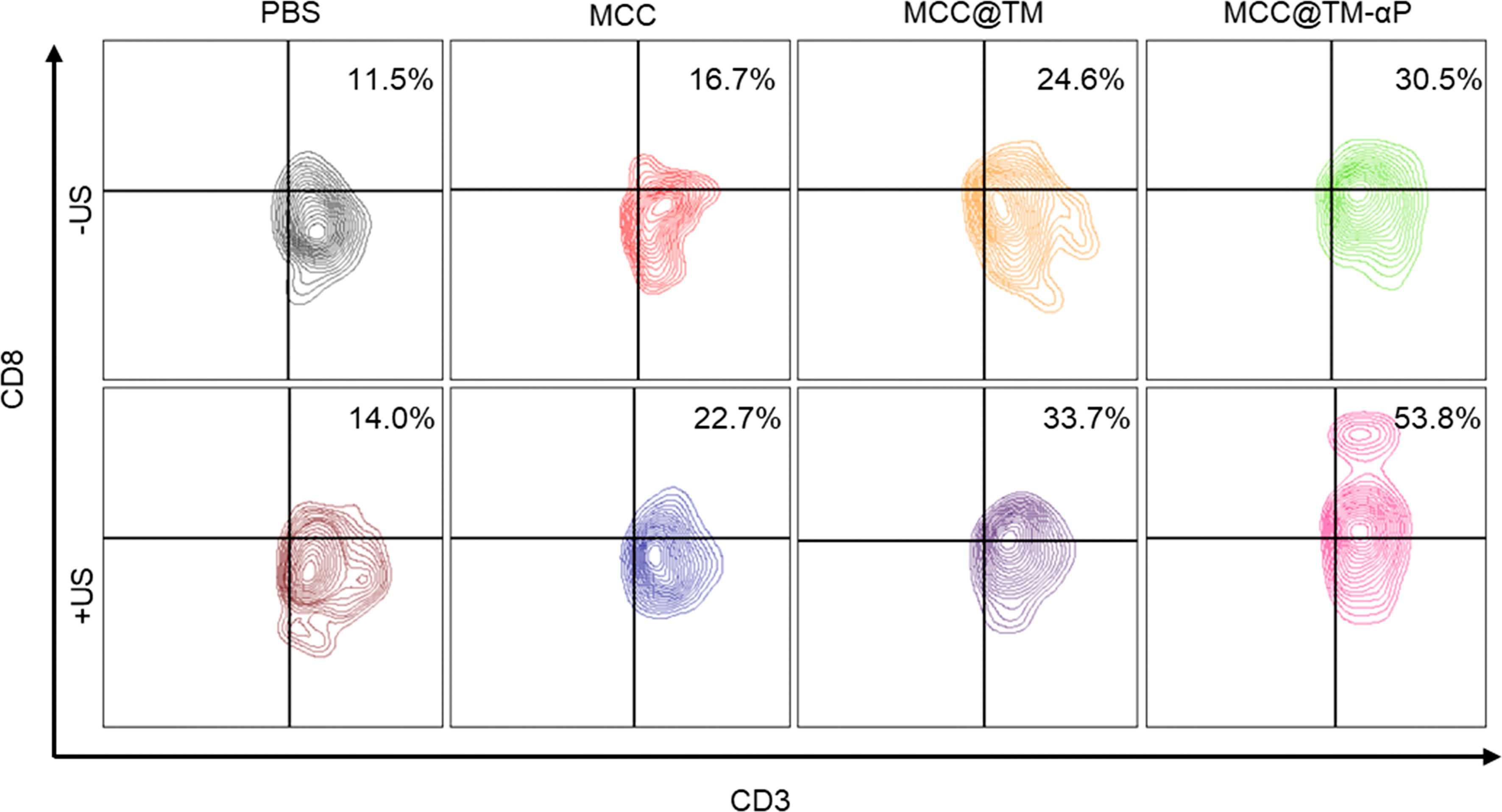


**Figure S13.** Flow cytometry assay of CD3^+^CD8^+^ T cells in primary tumors of mice in various treatment groups.

**
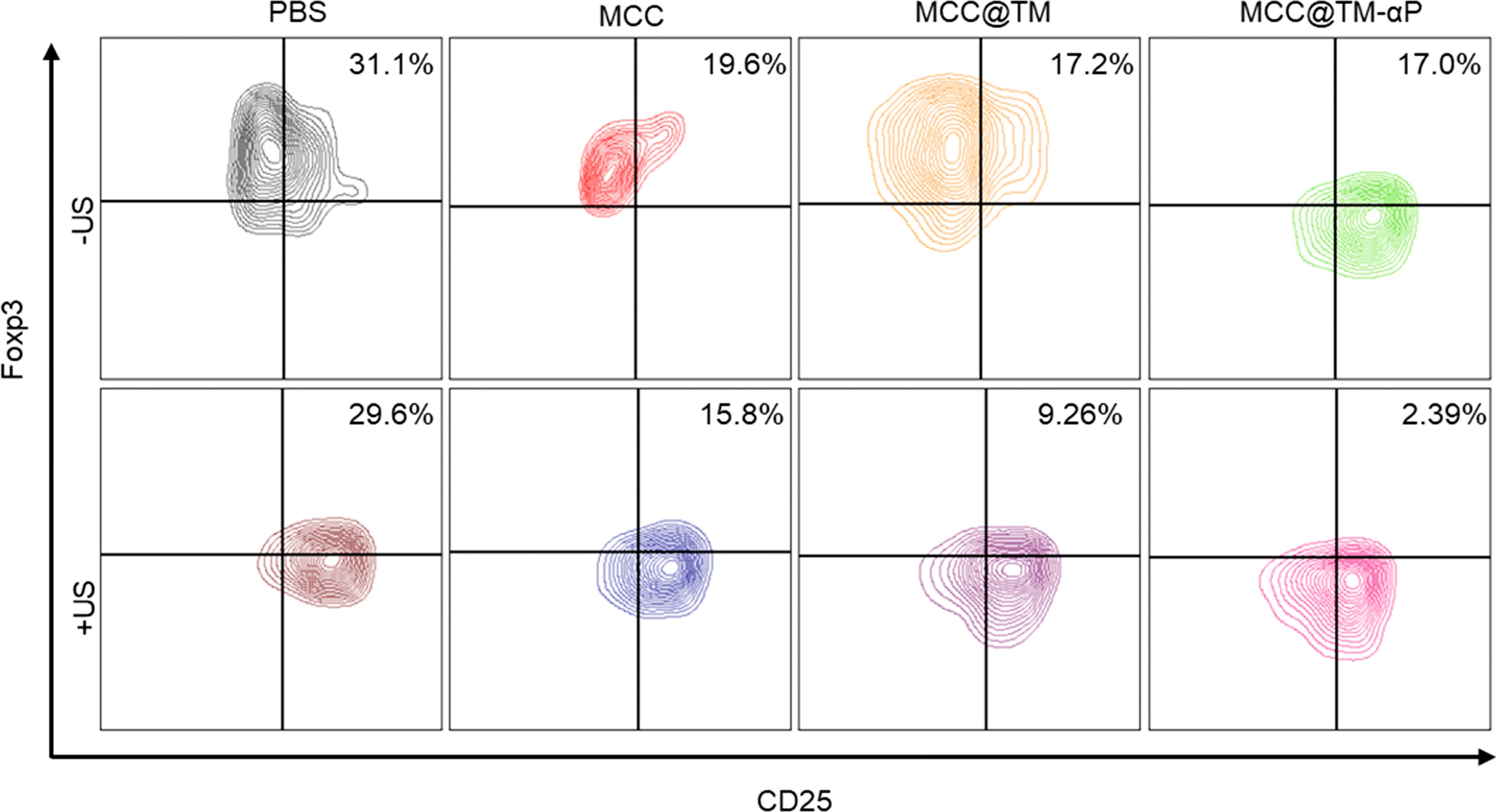
**

**Figure S14.** Flow cytometry assay of T_reg_ cells in primary tumors of mice in various treatment groups.


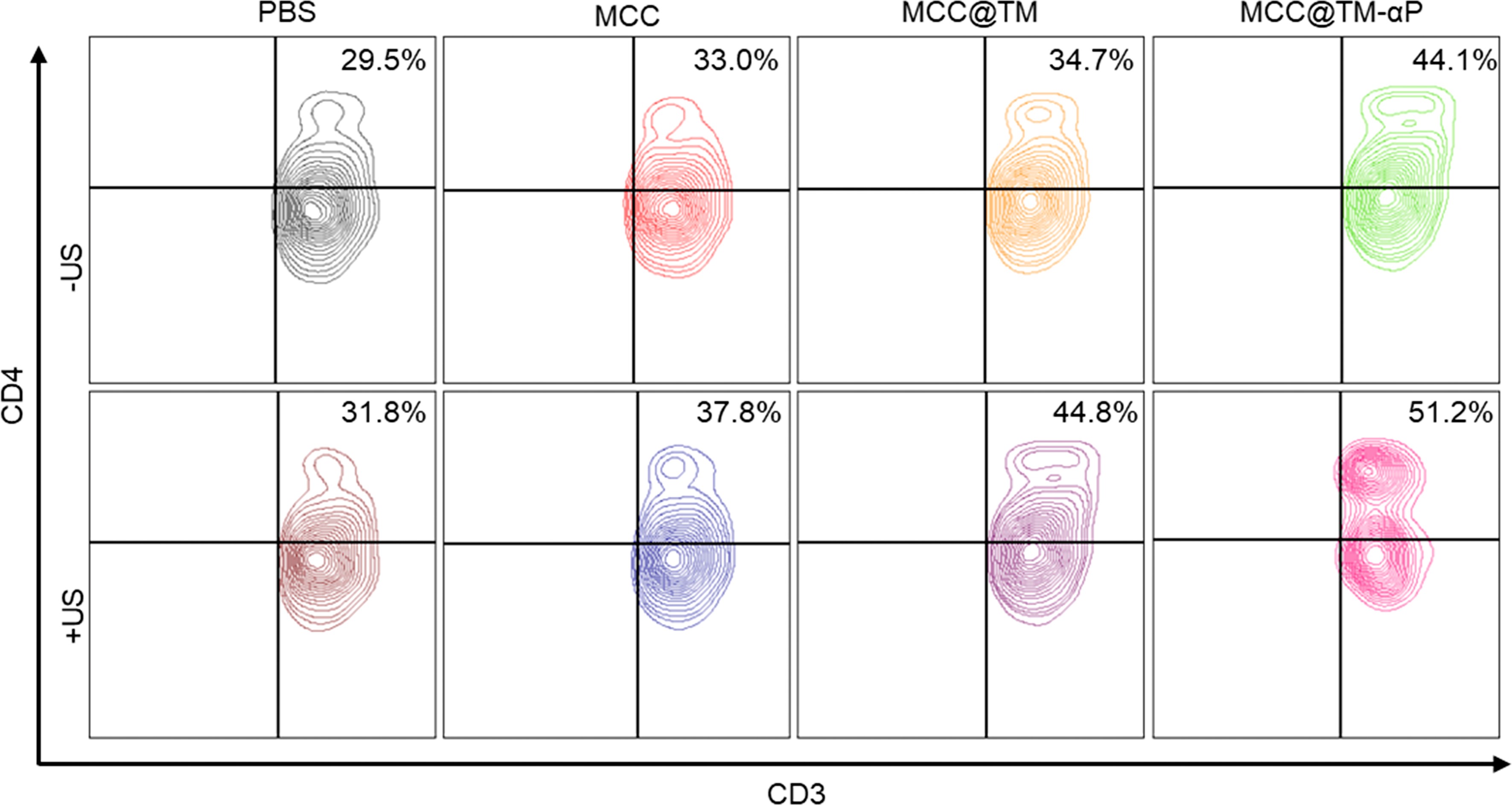


**Figure S15.** Flow cytometry assay of CD3^+^CD4^+^ T cells in distant tumors of mice in various treatment groups.


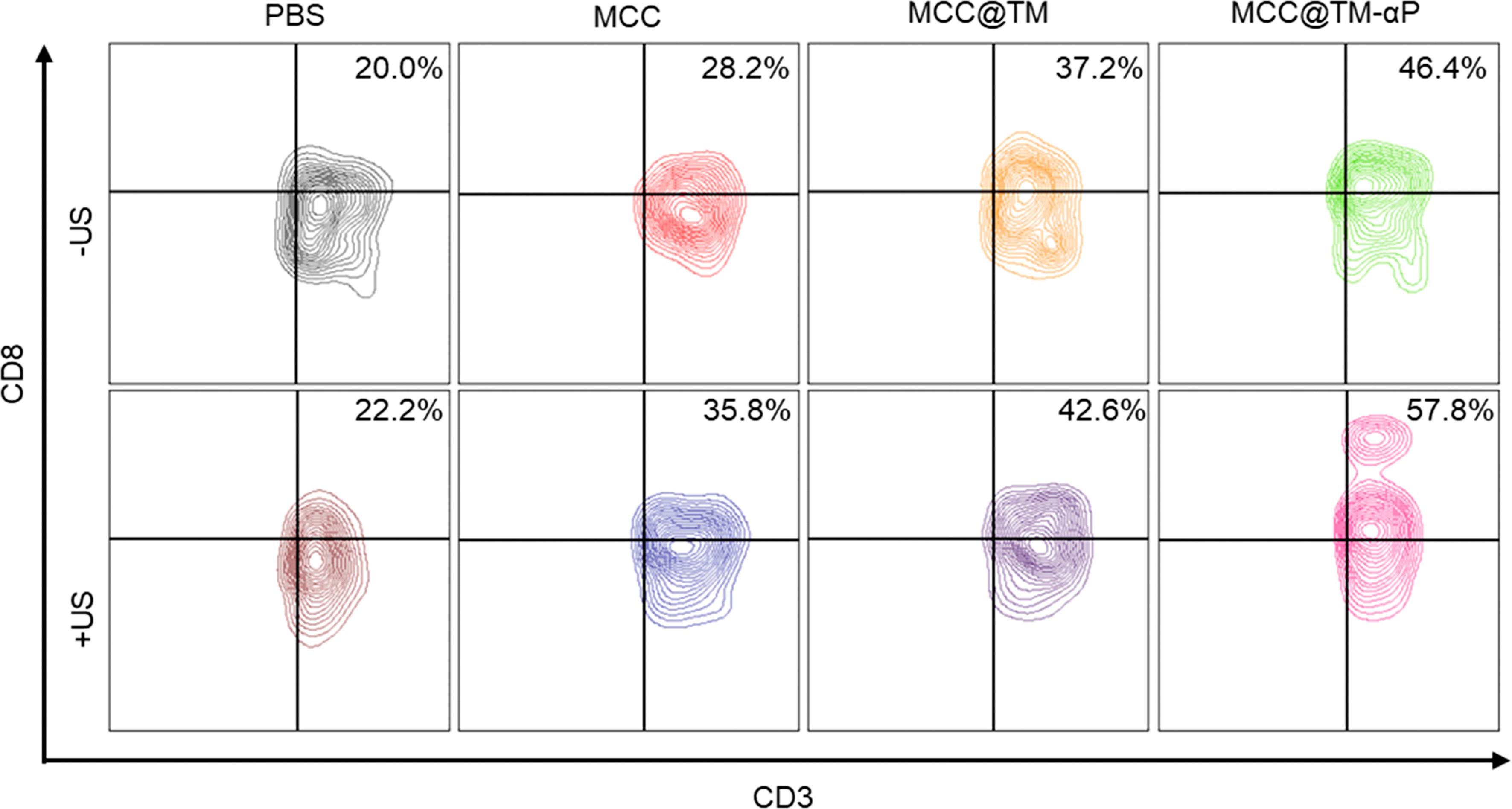


**Figure S16.** Flow cytometry assay of CD3^+^CD8^+^ T cells in distant tumors of mice in various treatment groups.

**
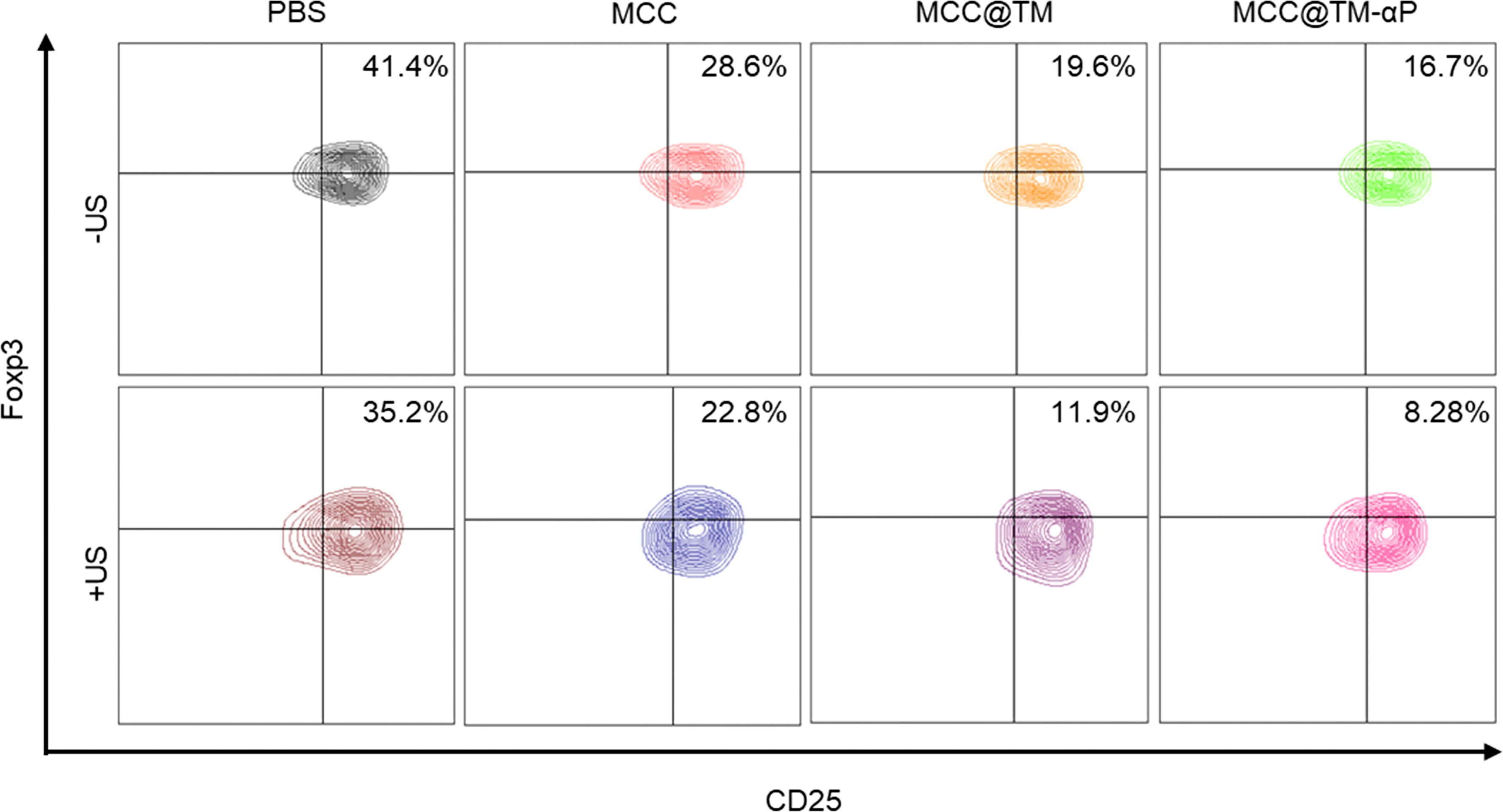
**

**Figure S17.** Flow cytometry assay of T_reg_ cells in distant tumors of mice in various treatment groups.

# 2. Experimental Section

*Material sources:* **CaCl_2_, KMnO_4_ and** bovine serum albumin (BSA) was provided by Macklin (Canada). Chlorin e6 (Ce6) was purchased from Yuanye Biotechnology Co., Ltd (China). Cystamine were provided by Sigma-Aldrich (USA). αPD-L1 antibody was purchased from BioXcell (USA). Cell counting kit-8 (CCK-8) were provided by Molecular Probes Inc. (USA). All other chemical reagents were purchased from Sinopharm Chemical Reagent Co., Ltd. (China). All immunofluorescent antibodies were purchased from BD Biosciences (USA).

*Materials characterization:* The material morphology was characterized using transmission electron microscopy (TEM). Particle size distribution, zeta potential, and colloidal stability were all measured using the Malvern Panalytical Zetasizer Nano ZS model ZEN3600 (Malvern Panalytical, UK). UV-Vis absorption spectra were recorded using a Shimadzu UV-3600 spectrophotometer (Japan) at room temperature, with a scanning range of 200-800 nm. Fluorescence properties were measured using a Shimadzu RF-6000 Fluorescence Spectrophotometer (Japan) at room temperature, with excitation wavelengths set according to the fluorescence properties of different nanoparticles. The protein contents in various materials was quantified using a BCA protein assay kit.

*Cell viability assessment:* 4T1 breast cancer cells were cultured in medium containing MCC, MCC@TM and MCC@TM-αP (25-400 μg/mL) for 24 h, and the cell viability measurement was conducted using CCK-8 assay.
